# Population coverage of artemisinin-based combination treatment in children younger than 5 years with fever and *Plasmodium falciparum* infection in Africa, 2003–2015: a modelling study using data from national surveys

**DOI:** 10.1016/S2214-109X(17)30076-1

**Published:** 2017-04

**Authors:** Adam Bennett, Donal Bisanzio, Joshua O Yukich, Bonnie Mappin, Cristin A Fergus, Michael Lynch, Richard E Cibulskis, Samir Bhatt, Daniel J Weiss, Ewan Cameron, Peter W Gething, Thomas P Eisele

**Affiliations:** Malaria Elimination Initiative, Global Health Group, University of San Francisco, San Francisco, CA, USA; Oxford Big Data Institute, Li Ka Shing Centre for Health Information and Discovery, Nuffield Department of Medicine, University of Oxford, Oxford, UK; Center for Applied Malaria Research and Evaluation, Tulane University School of Public Health and Tropical Medicine, New Orleans, LA, USA; Oxford Big Data Institute, Li Ka Shing Centre for Health Information and Discovery, Nuffield Department of Medicine, University of Oxford, Oxford, UK; World Health Organization, Geneva, Switzerland; World Health Organization, Geneva, Switzerland; US Centers for Disease Control, Atlanta, GA, USA; World Health Organization, Geneva, Switzerland; Oxford Big Data Institute, Li Ka Shing Centre for Health Information and Discovery, Nuffield Department of Medicine, University of Oxford, Oxford, UK; Oxford Big Data Institute, Li Ka Shing Centre for Health Information and Discovery, Nuffield Department of Medicine, University of Oxford, Oxford, UK; Oxford Big Data Institute, Li Ka Shing Centre for Health Information and Discovery, Nuffield Department of Medicine, University of Oxford, Oxford, UK; Oxford Big Data Institute, Li Ka Shing Centre for Health Information and Discovery, Nuffield Department of Medicine, University of Oxford, Oxford, UK; Center for Applied Malaria Research and Evaluation, Tulane University School of Public Health and Tropical Medicine, New Orleans, LA, USA

## Abstract

**Background:**

Artemisinin-based combination therapies (ACTs) are the most effective treatment for uncomplicated *Plasmodium falciparum* malaria infection. A commonly used indicator for monitoring and assessing progress in coverage of malaria treatment is the proportion of children younger than 5 years with reported fever in the previous 14 days who have received an ACT. We propose an improved indicator that incorporates parasite infection status (as assessed by a rapid diagnostic test [RDT]), which is available in recent household surveys. In this study we estimated the annual proportion of children younger than 5 years with fever and a positive RDT in Africa who received an ACT in 2003–15.

**Methods:**

Our modelling study used cross-sectional data on treatment for fever and RDT status for children younger than 5 years compiled from all nationally available representative household surveys (the Malaria Indicator Surveys, Demographic and Health Surveys, and Multiple Indicator Cluster Surveys) across sub-Saharan Africa between 2003 and 2015. Estimates for the proportion of children younger than 5 years with a fever within the previous 14 days and *P falciparum* infection assessed by RDT who received an ACT were incorporated in a generalised additive mixed model, including data on ACT distributions, to estimate coverage across all countries and time periods. We did random effects meta-analyses to examine individual, household, and community effects associated with ACT coverage.

**Findings:**

We obtained data on 201 704 children younger than 5 years from 103 surveys (22 MIS, 61 DHS, and 20 MICS) across 33 countries. RDT results were available for 40 of these surveys including 40 261 (20%) children, and we predicted RDT status for the remaining 161 443 (80%) children. Our results showed that ACT coverage in children younger than 5 years with a fever and *P falciparum* infection increased across sub-Saharan Africa in 2003–15, but even in 2015, only 19.7% (95% CI 15.6–24.8) of children younger than 5 years with a fever and *P falciparum* infection received an ACT. In meta-analyses, children younger than 5 years were more likely to receive an ACT for fever and *P falciparum* infection if they lived in an urban area (*vs* rural area; odds ratio [OR] 1.18, 95% CI 1.06–1.31), had household wealth above the national median (*vs* wealth below the median; OR 1.26, 1.16–1.39), had a caregiver with any education (*vs* no education; OR 1.31, 1.22–1.41), had a household insecticide-treated net (ITN; *vs* no ITN; OR 1.21, 1.13–1.29), were older than 2 years (*vs* ≤2 years; OR 1.09, 1.01–1.17), or lived in an area with a higher mean *P falciparum* prevalence in children aged 2–10 years (OR 1.12, 1.02–1.23). In the subgroup of children for whom treatment was sought, those who sought treatment in the public sector were more likely to receive an ACT (*vs* the private sector; OR 3.18, 2.67–3.78).

**Interpretation:**

Despite progress during the 2003–15 malaria programme, ACT treatment for children with malaria remains unacceptably low. More work is needed at the country level to understand how health-care access, service delivery, and ACT supply might be improved to ensure appropriate treatment for all children with malaria.

**Funding:**

US President's Malaria Initiative and Medicines for Malaria Venture.

## Introduction

Nearly half of the world's population is at risk of *Plasmodium falciparum* malaria,^1^ the most lethal species of malaria parasite. In 2015, *P falciparum* infections led to an estimated 187 million cases and 398 000 deaths across sub-Saharan Africa.^1,2^ Fortunately, *P falciparum* infection is treatable with available antimalarials. Following the emergence of widespread drug resistance to chloroquine and sulfadoxine–pyrimethamine,^3^ artemisinin-based combination therapies (ACTs) were introduced as a highly effective treatment for uncomplicated malaria (both for *P falciparum* and *Plasmodium vivax* infections), preventing progression to severe disease and death.^4,5^

The WHO-recommended first-line treatment for uncomplicated *P falciparum* malaria in nearly all endemic countries is an ACT.^6^ Funding for procuring ACTs has greatly increased and they are now widely available from multiple manufacturers and in many formulations. Between 2003 and 2007, nearly all countries in Africa changed their treatment policy to ACTs as the first-line treatment of uncomplicated malaria, and since then, a major rise has been seen in global ACT procurement and distribution.^7^

To date, one of the most commonly used indicators for monitoring global progress in the treatment of malaria has been the proportion of children with a fever in the previous 2 weeks that received an effective antimalarial, as measured by mother's recall during nationally representative household surveys.^8^ Although this measure is available from household surveys and gives some indication of the extent of malaria treatment, it has several notable limitations. Most crucially, the proportion of children who have had an antimalarial does not indicate whether the fever was associated with an actual malaria parasite infection. Even if a country achieves a reasonably high level of treatment of fevers with an effective antimalarial, which for the past decade has been an ACT, this measure can be highly misleading because it includes inappropriate treatment of non-malarial fevers. Although an increasing proportion of patients with suspected malaria undergo malaria diagnostic testing, in many settings across Africa, treatment still relies on clinical diagnosis of malaria on the basis of fever without laboratory confirmation. Another limitation of this approach is that household surveys are done intermittently, or not at all in some countries. Because of changes in the availability of drugs or clinical practice, treatment coverage might vary in the years between surveys. Providing an estimate of treatment coverage in non-survey years might help programmes better assess their progress and respond in a more timely manner to deficiencies in service delivery.

A more useful indicator for measuring progress in combating malaria is the proportion of children with a fever plus a *P falciparum* infection in the previous 2 weeks who received an ACT. Since 2006, many national household surveys in Africa have added in assessments using rapid diagnostic tests (RDTs) that detect the *P falciparum* parasite histidine-rich protein 2 (hrp2), which can be used as a measure of *P falciparum* parasite prevalence. Because hrp2 circulates for up to several weeks after *P falciparum* has been cleared,^9,10^ RDTs can detect infections up to 42 days after parasite clearance, with almost all infections detected up to 7 days, and about 80% detected up to 14 days after parasite clearance.^11^ Because of this property, hrp2 RDTs done at the time of the survey provide an approximate measure of 2-week infection period prevalence^12^ that overlays effectively with the reported history of fever and malaria treatment in the surveys.

In this study, our objective was to estimate the proportion of children younger than 5 years with a recent fever and a positive *P falciparum* RDT who were treated with an ACT from 2003 to 2015 in all *P falciparum-*endemic countries in Africa, as measured by national household surveys.

## Methods

### Study design and data sources

This study was a modelling analysis that used cross-sectional data obtained from nationally representative household surveys. We used the STROBE guidelines in the reporting of this study,^13^ and a checklist is included in the appendix pp 38–39.

We included data from publicly available national population-based surveys that measured the 2-week history of fever and malaria treatment for children younger than 5 years, categorised by type of antimalarial received. These surveys were the Malaria Indicator Surveys (MIS), Demographic and Health Surveys (DHS), and Multiple Indicator Cluster Surveys (MICS) done in 2003–15. The sampling methods for these surveys have been described in detail elsewhere.^14^ In addition to a questionnaire intended to measure household wealth, household insecticide-treated net (ITN) ownership, and caregiver education level, these surveys also ascertain data on occurrence of and care for fever, cough, and diarrhoea from all caregivers of children younger than 5 years, and the MIS and some DHS measure *P falciparum* infection status of children younger than 5 years in sampled households.

We obtained data on the number of ACTs and other antimalarials distributed by national programmes per year in 2003–15 from the WHO World Malaria Report.^7^ We standardised ACT distributions by dividing by the total population at risk per country (using estimates from the Malaria Atlas Project [MAP]) to produce ACT availability per capita (ACT_cap_), and calculated annual population estimates using growth rates derived from the UN Population Prospects database. We also obtained annual estimates of *P falciparum* prevalence in children aged 2–10 years (*Pf*PR_2–10_) from MAP that we extracted at the latitude and longitude for each survey cluster. We excluded countries that had a national mean *Pf*PR_2–10_ of less than 2% in any year, because the prevalences of *P falciparum* and fever were too low to provide reasonable sample sizes in these countries.

### Defnitions

We defined coverage of ACT treatment for children younger than 5 years with fever and a *P falciparum* infection as the proportion of children younger than 5 years with a fever in the previous 2 weeks and a positive RDT test at the time of survey who were reported as receiving an ACT. We defined household ITN ownership as the presence of at least one ITN in the household at the time of the survey, and determined household wealth using principal components analysis of household assets.^15^ Education level of the child's caregiver was categorised as any or none. Endemicity level was defined for each survey cluster as hypoendemic (<10% *Pf*PR_2–10_), mesoendemic (10–50%), and hyperendemic or holoendemic (>50%) on the basis of the mean *Pf*PR_2–10_ at each cluster. Season was determined by whether the median survey date was during the rainy or dry season. We classified the source of treatment for fever as public if treatment was sought from any governmental source, and as private if treatment was sought from any nongovernmental source, including informal care. To examine regional trends, we classified countries on the basis of UN Africa subregion (western, central, and eastern), and whether or not they received funding through the Affordable Medicines Facility malaria (AMFm) scheme.

### Statistical analysis

In most surveys except the MIS and some recent DHS, parasite prevalence by RDT was not measured. Given the paucity of data available to create a complete time series measuring ACT treatment for children younger than 5 years with fever and a *P falciparum* infection across countries from 2003–15, we used a three-step modelling approach to predict coverage for surveys without RDT measurement and used approximate Bayesian time series methods that included ACT distribution data to impute data for countries and years with missing data. We used random effects meta-analyses of the survey datasets to assess associations between individual-level ACT treatment and various predictors. All analyses were done using R 3.2.3 and Stata 13.1.

### National-level and continent-level coverage estimates

First, for national surveys without RDTs (most DHS and all MICS), we modelled the propensity of children with a fever to be RDT positive on the basis of a set of predictive factors. We used all available survey datasets with RDT results at the time of survey to parameterise a logistic regression model to predict malaria parasite infection in febrile children. In the regression model, we included the child's age, household wealth quintile, household ITN ownership, urban or rural status, season (rainy or dry), and malaria transmission intensity for the survey year, measured by logit-transformed *Pf*PR_2–10_. To account for uncertainty and obtain predictions of RDT status in all children with a fever from the compiled surveys, we sampled values of logit-transformed *Pf*PR_2–10_ at each survey location and time, and used the coefficients estimated from logistic model estimations to produce predicted probabilities of RDT status for each child. We then sampled values using the binomial distribution to produce binary predictions for each child (appendix p 4). For each of these child-level predictions we then calculated the national survey-weighted proportion of children younger than 5 years with a fever and positive RDT test (as measured, or predicted if not measured) who received an ACT for each survey.

At the last step, we imputed ACT coverage values for each country and year with no survey dataset available using a generalised additive mixed model (GAMM) that incorporated the relationship between ACT coverage and ACT_cap_ across countries. National annual ACT coverage was modelled as a function of time, country, UN Africa subregion, and ACT_cap_. Gaps in the ACT coverage time series for each country were filled on the basis of posterior means of the model's fixed effect, using known values as anchor points (appendix pp 11–12).

Continental-level estimates were weighted by the population at risk for each country. We produced separate estimates of ACT coverage by UN Africa subregion, whether or not they were part of the AMFm scheme, residence (urban or rural), socioeconomic status (above or below country median), endemicity level (hypo endemic, mesoendemic, or hyperendemic or holo endemic), and health-care provider (public or private). We also produced estimates of ACT coverage in children younger than 5 years with a fever and a negative RDT result.

### Meta-analyses of factors influencing individual-level coverage

Finally, we used survey datasets with both measured and predicted RDT status to assess the association between treatment with an ACT for children with fever and *P falciparum* infection and individual, household, and community-level factors using random effects meta-analysis. Urban or rural, wealthy or poor, mean (logit-transformed) *Pf*PR_2–10_, household ITN ownership, caregiver education (any or none), and child's age (>2 years or ≤2 years) were first included in multivariable models for each survey dataset with a random effect at the primary sampling unit level. The coefficient and SE for each factor from these meta-analyses was then entered into a separate meta-regression for each factor, with a random effect at the survey level. We ran the same models including the health-care provider type, restricted to those children who sought treatment, and additionally a meta-regression model among all children with fever, including only survey-level associations between ACT treatment and RDT status and a covariate for before and after 2012 to assess the effect of RDT status on ACT coverage after policy changes in 2010.

### Model validation

We assessed the models for RDT predictions, ACT coverage, and ACT availability by withholding a sample of the observed values as a testing dataset, and running the prediction process on the remaining training dataset. We assessed predictive accuracy using both individual-level hold-out samples and hold-out samples of entire surveys. We compared predicted values with observed values and assessed the accuracy of predictions using the area under the curve (AUC) statistic for RDT status, and the root mean square error (RMSE), mean absolute error, and mean absolute scaled error for ACT coverage.

To assess how a shorter time period (<14 days) between survey and fever date affected our results, we did a sensitivity analysis in which the recall period of a fever was limited to within the previous 2–7 days. This analysis was possible only in a subset of 12 MIS surveys and one DHS survey that measured the days since the fever started.

### Role of the funding source

The funders of the study had no role in study design, data collection, data analysis, data interpretation, or writing of the report. All authors had full access to all the data in the study and had final responsibility for the decision to submit for publication.

## Results

**Figure 1:**
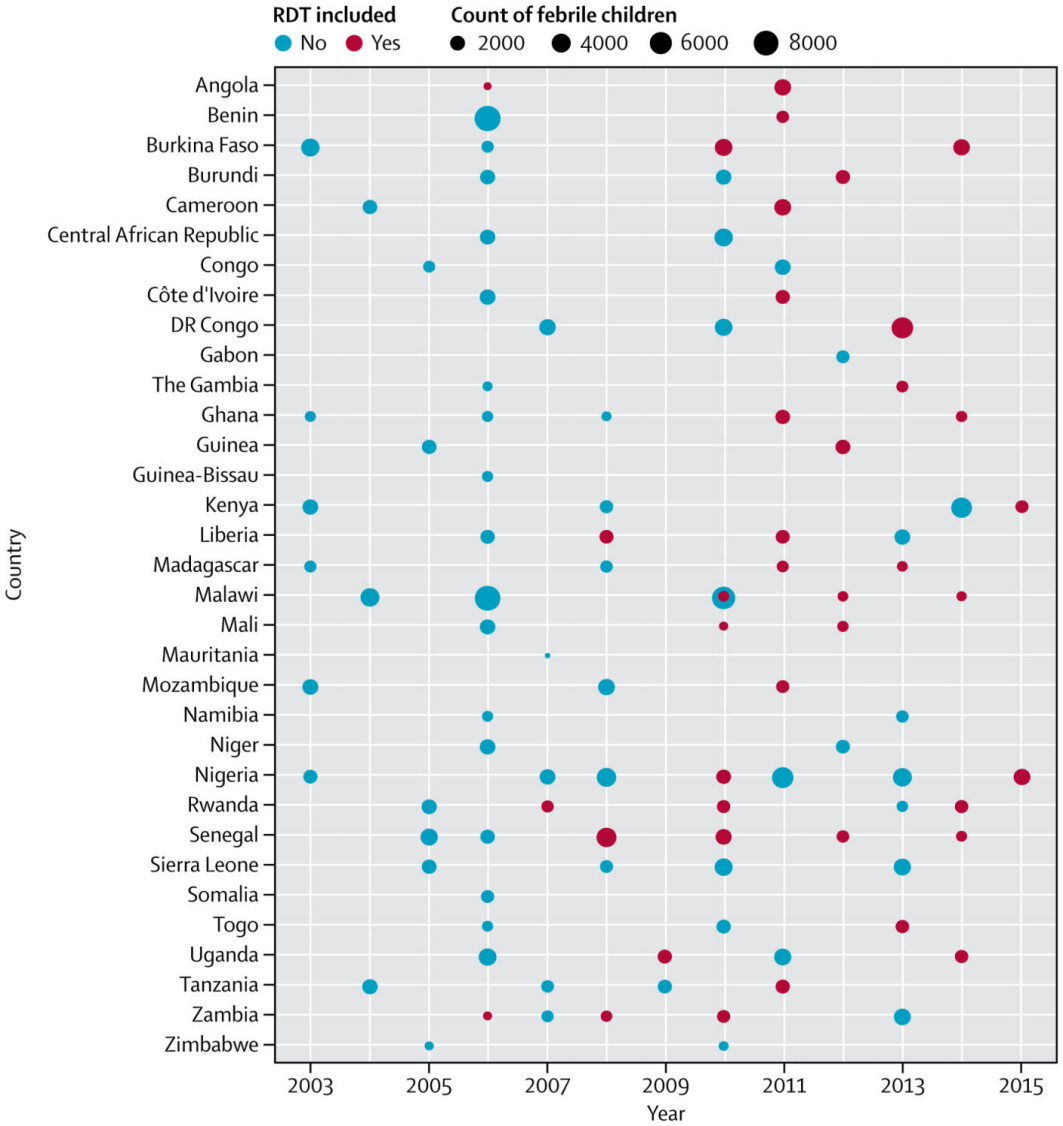
Frequency plot of surveys included in study, by country and year, with count of febrile children younger than 5 years and whether RDT data were collected in survey , 103 surveys were included (22 MIS, 61 DHS, and 20 MICS), of which 40 collected RDT data (19 MIS, 20 DHS, and one MICS). DHS=Demographic and Health Survey. MICS=Multiple Indicator Cluster Survey. MIS=Malaria Indicator Survey. RDT=rapid diagnostic test.

The final dataset included information on 201 704 children younger than 5 years from 103 surveys (22 MIS, 61 DHS, and 20 MICS) across 33 countries in sub-Saharan Africa from 2003–15. RDT results were available for 40 of these surveys including 40 261 children (20%; figure 1), and we predicted RDT status for the remaining 161 443 children (80%). RDT results were far more commonly available in recent surveys: 33 (63%) of 52 surveys done from 2010 onwards collected RDT data, compared with only seven (14%) of 51 before 2010.

In the RDT-status prediction model, increasing age, decreasing wealth, no household ITN ownership, increasing cluster *Pf*PR_2–10_, rural location, and survey being done during the rainy season were all strongly associated with a positive RDT (table 1). This model achieved relatively good predictive accuracy for individual RDT status, with a mean AUC of 0.78 from 100 independent 15% hold-out samples. Survey-level predictive accuracy varied from 0.59 to 0.86 (appendix p 7), with a mean of 0.73, and ACT coverage using predicted RDT status was consistent with ACT coverage using observed RDT status (appendix p 6). The GAMM model for predicting missing country years achieved an RMSE of 7.4. Further validation results are presented in the appendix (p 12).

**Figure 2:**
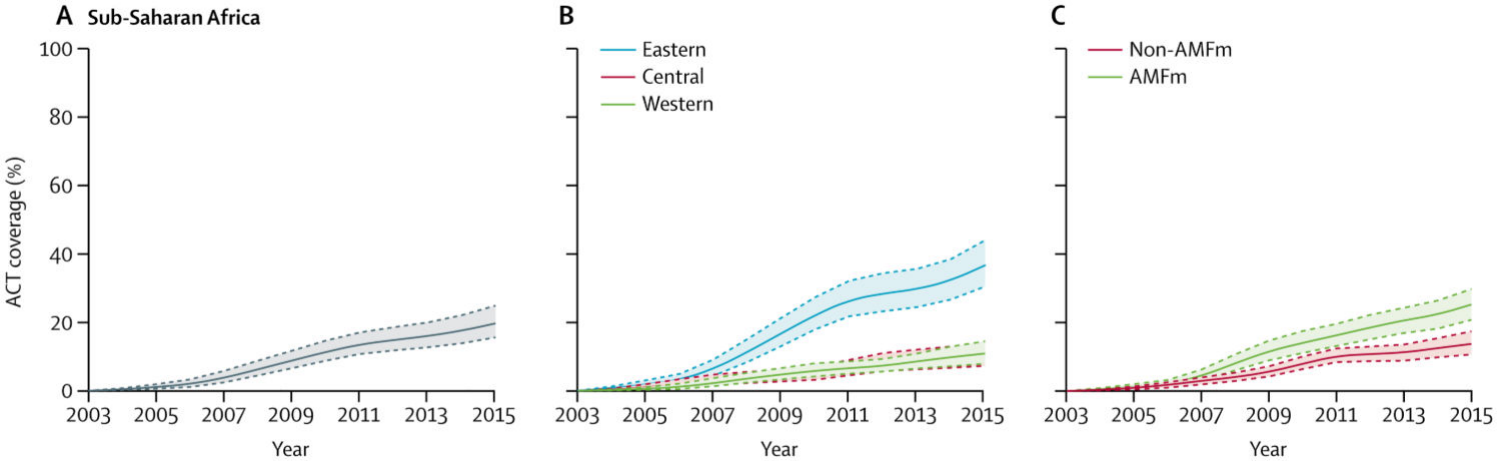
Proportions of children younger than 5 years with fever and *Plasmodium falciparum* infection who received an ACT in sub-Saharan Africa, 2003–15 ACT coverage for sub-Saharan Africa (A), stratified by UN subregion (B), and by presence or absence of an AMFm scheme (C). Southern Africa is not included in the graph because all countries other than Namibia had a mean *P falciparum* prevalence in children aged 2–10 years of less than 2%. Namibia was included in central Africa because its endemic area is at the same latitude as countries in this region. Dotted lines show 95% CI. ACT=artemisinin-based combination therapy. AMFm=Affordable Medicines Facility, malaria.

**Figure 3:**
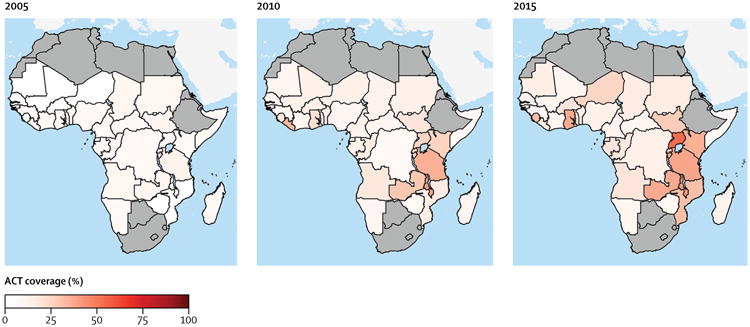
Proportions of children younger than 5 years with fever and *Plasmodium falciparum* infection who received an ACT, per country, in 2005, 2010, and 2015 , Countries with a mean *P falciparum* prevalence in children aged 2–10 years of less than 2% are in grey. ACT=artemisinin-based combination therapy.

The continent-wide proportion of children younger than 5 years with a fever plus *P falciparum* infection confirmed by RDT who received an ACT was 19.7% (95% CI 15.6–24.8) in 2015 (table 2). ACT coverage across sub-Saharan Africa increased gradually between 2005 and 2015, accelerating slightly between 2009 and 2011 (figure 2A). ACT coverage was significantly higher in eastern Africa than in central and western Africa, and no difference in coverage was seen between central and western Africa (figure 2B). Coverage was higher in AMFm countries than in non-AMFm countries from 2008 to 2015, with a slightly greater difference after 2011 (figure 2C). The proportion of *P falciparum*-infected children younger than 5 years with fever receiving an ACT in 2015 ranged from a low of 0.6% (95% CI 0.0–4.1) in Somalia to a high of 70.2% (65.6–74.5) in Uganda (exact country-level data not shown; figure 3).

**Figure 4:**
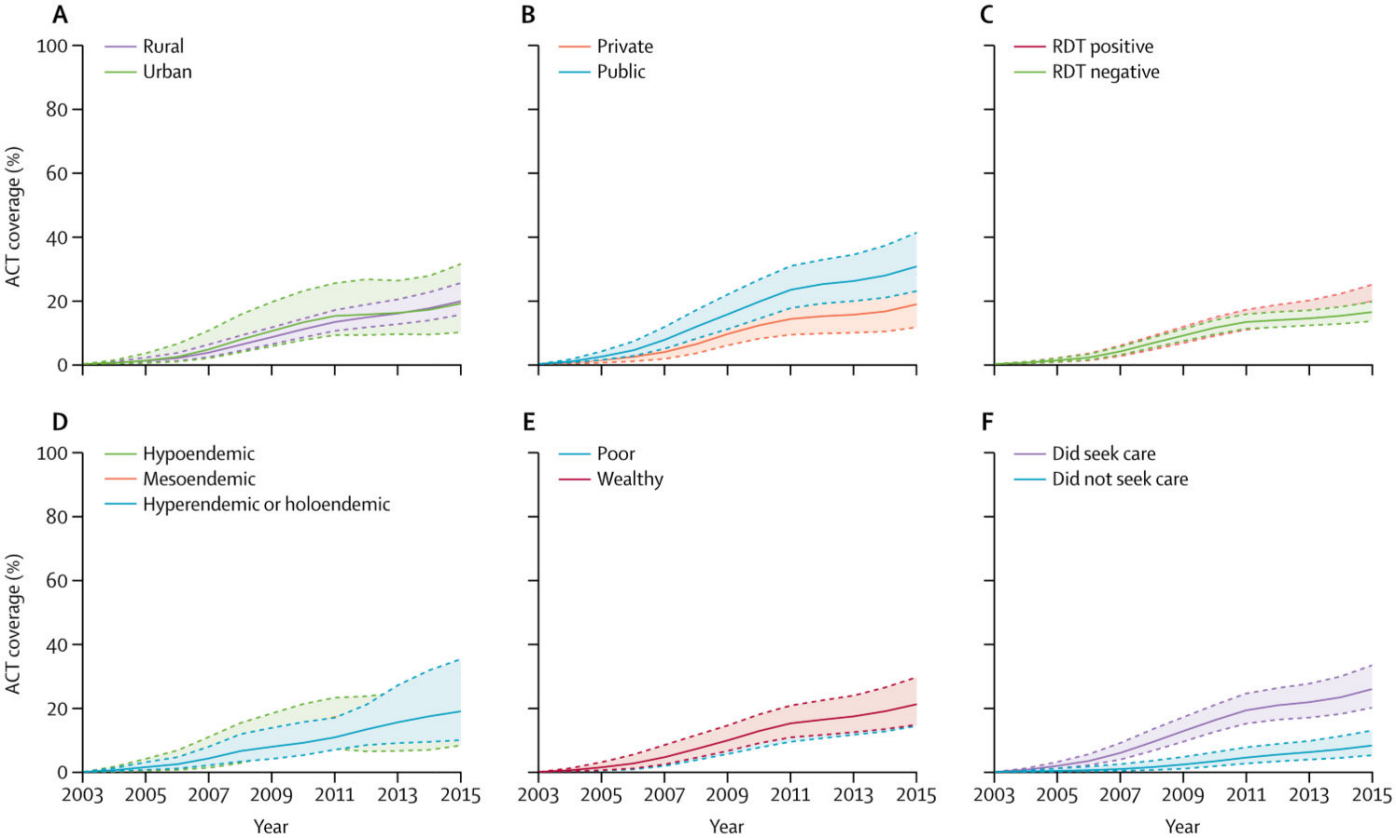
Proportions of children younger than 5 years with fever and a *Plasmodium falciparum* infection in sub-Saharan Africa who received an ACT in 2003–15, stratified by demographic and clinical variables , ACT coverage stratified by residence type (A), public or private health-care provider (B), positive or negative RDT results (C), malaria endemicity (D), wealth relative to median country wealth index (E), and treatment seeking (F). ACT=artemisinin combination therapy. RDT=rapid diagnostic test.

Continent-wide ACT coverage of children with fever plus *P falciparum* infection was similar irrespective of location of residence, household wealth, and malaria endemicity (figure 4); ACT coverage in 2015 was higher in the subgroup of children for whom health care was sought than for those for whom health care was not sought (table 2). Coverage for children who had treatment at public providers was almost twice that of those who used private providers in 2015 (figure 4, table 2). Continent-wide ACT coverage for children whose RDTs were positive or negative was roughly the same between 2003 and 2012, with coverage appearing to be slightly higher for those with positive RDT results after 2012 (figure 4).

In a meta-analysis of country datasets, children with a fever and *P falciparum* infection were more likely to receive an ACT if their caregiver had any education, if their household wealth was above the country median, if their household owned an ITN, and if they lived in an urban area (table 3). Of the children for whom treatment was sought, children older than 2 years, whose caregiver had any education, whose household owned an ITN, who were living in a community with higher mean *Pf*PR_2–10_, or for whom treatment was sought at a public facility were more likely to receive an ACT (table 3). Finally, a positive RDT status in children with fever was far more likely to be associated with receiving an ACT in 2012–15 than it was in 2003–11 (OR 1.35, 95% CI 1.15–1.58).

## Discussion

In this study we estimated the proportion of children younger than 5 years with a fever and *P falciparum* infection confirmed by RDT receiving an ACT by country and year for 2003–15 across sub-Saharan Africa. To the best of our knowledge, this study is the first to use this indicator for assessing the progress of malaria treatment in children who need treatment, at the continental and country level and by year. Although we documented noticeable increases in ACT coverage from 2003 to 2015, which correspond with increased investments in ACTs and RDTs, coverage remains unacceptably low across Africa: over three-quarters (80.3%) of children with malaria did not get a potentially life-saving ACT in 2015. The two biggest drivers of this coverage gap are poor access to and delivery of health services. Only 8.3% of people who did not seek care from a health provider outside the home received an ACT, and even of those who did seek care, only a quarter received an ACT; of these individuals, those who sought treatment in the public sector were far more likely to receive an ACT than those who went to a private health-care provider.

Considerable variation was seen in coverage across countries and over time, and a few large countries with low coverage—eg, Nigeria—strongly affected regional and continental estimates. These low-coverage estimates, and especially the low estimates in some large, high-burden countries, show that people most in need in highly endemic areas are often the least likely to get appropriate treatment for malaria. Notably, the greatest improvement in coverage was seen mainly in eastern African countries that have also shown progress in scaling up ITN coverage,^16^ suggesting that higher ACT coverage is linked to overall scale-up of malaria interventions. Greater improvement in coverage was also seen in countries supported by AMFm funding, although we were unable to determine from this analysis whether this effect was directly associated with the total amount or type of funding, or some other factor.

Addressing disparities in access to and delivery of ACTs is crucial for preventing severe disease and death in those most in need, and might also contribute to the reduction of transmission when high population coverage is reached.^2,17^ Although in continental-level estimates, the CIs for ACT coverage stratified by urban or rural residence, wealth, endemicity, and RDT status overlapped, in individual-level meta-analyses we found that poorer children living in rural areas with fever and a *P falciparum* infection were significantly less likely to receive an ACT than children who lived in urban areas and had a household wealth index above the national median, as were those whose caregiver had no education and whose household did not own an ITN. These variables—area of residence, household wealth, education, and ITN ownership—are all proxies for access to health services and influence the seeking of treatment for fever. Estimates of the proportion of children younger than 5 years with fever taken for any care are between 56% and 69% across sub-Saharan Africa.^18^ Of children for whom treatment was sought, urban residence and wealth were not significant predictors of ACT treatment. Improving access to care and provision of ACT treatment in poor, rural populations^19^ is crucial for sustaining efforts to control malaria and progressing towards elimination. Our finding that 8.3% of children received an ACT even when their caregivers did not report seeking treatment outside the home was somewhat surprising, but largely driven by data from countries with very high overall ACT coverage (eg, Uganda) or where mass drug administration activities have recently been done (eg, Zambia). In these cases, there might be large quantities of circulating ACTs in the community.

Our findings are consistent with those of other studies that have explored the combination of patient and provider factors that influence ACT coverage. Galactionova and colleagues^20^ documented large differences between the factors that most influenced effectiveness of malaria case management across sub-Saharan Africa, including access to a health-care provider, provider compliance and patient adherence with front-line antimalarial policy, and drug quality. Consistent with our analysis, they found that in many countries effective coverage was low because of poor access to heath-care providers, but in several countries with high access to providers, effective coverage was low because of poor provider compliance with national ACT policy.^20^ Littrell and colleagues^21^ similarly found low effective coverage in Zambia due to a combination of low treatment-seeking behaviour by patients and low rates of diagnostic confirmation at health facilities.^21^ Several similar studies have documented individual provider barriers, including frequent stockouts^22^ and low availability of diagnostics, and individual patient barriers such as distance to formal facilities, lack of financial resources, and low maternal education.^23,24^

The private sector continues to lag behind the public sector at scaling up RDTs and ACTs, showing a huge unmet need in treatment because in much of sub-Saharan Africa, rural, poor households receive care primarily through the private sector.^18,24–26^ We found substantially lower coverage for children who sought care at private providers than for those who visited public providers in both continent-level predictions and individual-level regression analyses, which is consistent with previous research.^27^ Of children with fever and a *P falciparum* infection who sought treatment in the private sector, less than a fifth received an ACT in 2015. Substantial efforts have been made to increase the availability of ACTs through the private sector in Africa through initiatives like the AMFm, with notable improvements in some areas,^28–30^ but the scarce access to ACTs through the private sector remains an important impediment to malaria control and burden reduction, especially for people living in rural areas, who have below-average household wealth.

The traditional indicator of ACT coverage, measured in all children with fever, does not allow for monitoring and assessment of appropriate treatment for those who need it. Instead, the improved indicator of ACT coverage that includes RDT status allows assessment of not only the proportion of children in need who received ACT, but also the proportion without a malaria parasite infection that inappropriately received ACT. We found only minor differences in ACT coverage between children with a malarial fever and those with a non-malarial fever, which points to considerable inefficiencies in ACT allocation. This result is not entirely surprising because RDTs were not widely scaled up across most of Africa until after 2010, and most suspected cases were treated on the basis of clinical diagnosis only. After 2012, our results show that malaria parasite-infected children with fever were more likely to receive an ACT than children with a fever but no malaria infection, suggesting improvements in appropriate treatment. Although we did not examine diagnostic coverage in this study, our results suggest that improvements are happening; however, on a continental scale, scale-up of diagnostics might not yet be having a marked effect on prescription practices and appropriate treatment.

The approach used in our study has some limitations. First, because of the time lag between measurement of RDT status and treatment for fever, a positive RDT result at the time of survey does not necessarily indicate that a child had a malaria infection during their recent fever episode and, if they were tested at a clinic, that they would have had a positive RDT result. Although all fevers combined with *P falciparum* infection warrant treatment with an ACT, some children who either were not yet infected at the time of their fever or had very low-density infections might have had negative RDT test results or been clinically diagnosed with a different condition. Similarly, a negative RDT at the time of survey does not rule out a *P falciparum* infection in recent weeks, especially given the relatively low sensitivity of particular RDTs in field settings^31^ and the possibility of hrp2 deletion in some parasites.^32^ Third, although predictive models showed good accuracy, we cannot be certain of accurately ascertaining each child's RDT status. However, children predicted to be RDT positive were more likely to be poor, have no household ITN, and live in high-prevalence and rural areas than those who were predicted to be RDT negative, all of which suggest greater need for ACT treatment for fever. Fourth, only a few surveys include the number of days since the fever was reported, which would have allowed us to remove children with very short or long times since fever, and reduced the potential for recall bias. Using those surveys that included this indicator, we did a sensitivity analysis that showed consistency in estimating ACT coverage in children with a fever and *P falciparum* infection using a shorter, 1 week period of recall. In a previous study,^33^ we documented high accuracy of caregiver recall for ACT treatment within the previous 2 weeks from a household survey in Zambia, but recall bias could be greater in other settings. Fifth, our predictions were limited by the relatively small sample sizes in many of the surveys, because these surveys are not typically powered to obtain representative estimates for children with fever, and also might not capture subnational heterogeneity in malaria transmission risk. Because the relatively small sample sizes probably influenced our ability to produce precise estimates, we incorporated multiple levels of uncertainty and reflect these in our estimates. Finally, the indicator of ACT treatment for fever and a positive RDT result is only a proxy measure for appropriate treatment for uncomplicated malaria. Although the inclusion of infection status is a notable improvement, appropriate treatment involves additional steps such as ensuring correct dosage and patient adherence, and exploring alternative causes of fever, which are not captured by this indicator. As countries move to improve data collection through the health system, robust routine collection of test results and treatment outcomes for fever is crucial for monitoring and evaluation of malaria case management; periodic health facility surveys that include exit interviews for patients seeking care for fever could provide an additional important datapoint to complement routine and household survey data.

We document, for the first time to our knowledge, continental and country-specific trends in ACT coverage in children who need an ACT (ie, those who have fever and a *P falciparum* parasite infection) over the past decade of scale-up, and show that even after the large investments in malaria prevention, diagnostic testing, and treatment over the past decade, ACTs are not reaching most children with malaria who need treatment. In addition to investing in new technologies and tools, malaria programmes need to ensure that individuals with malaria receive treatment through greater access to health care and consistent availability and provision of ACTs in areas of greatest need. Improved care in all health sectors, including continued scale-up of integrated community case management, will be essential for improving access to ACTs, especially for rural households with low incomes and low access to health services. This research shows the large gap in appropriate treatment of uncomplicated malaria and the potential to increase the positive impact of malaria control and elimination programmes.

## Supplementary Material

supplement

## Figures and Tables

**Table 1:** Coefficient values of model predicting rapid diagnostic test status for children younger than 5 years

	Coefficient	95% CI	p value
Age (years)			
<1	Ref	..	..
1-2	1.62	1.49-1.75	<0.0001
2-3	2.36	2.18-2.56	<0.0001
3-4	2.77	2.55–3.02	<0.0001
4-5	2.88	2.64-3.14	<0.0001

Wealth quintile			
Poorest	Ref	..	..
Poor	1.06	1.00–1.13	0.0586
Middle	1.05	0.98-1.12	0.1849
Wealthy	0.82	0.76-0.88	<0.0001
Wealthiest	0.44	0.40-0.49	<0.0001

Urban residence	0.69	0.65-0.74	<0.0001

Household owns ≥1 insecticide-treated net	0.93	0.89-0.98	0.0080

*Pf*PR_2–10_	2.10	2.06-2.14	<0.0001

Survey done in rainy season	1.18	1.12-1.24	<0.0001

Estimates are based on a logistic model of 40 261 children younger than 5 years with rapid diagnostic test status. Using these coefficients, rapid diagnostic test status was then predicted for 161 443 children. *Pf*PR_2–10_=mean (logit-transformed) *Plasmodium falciparum* prevalence in children aged 2-10 years.

**Table 2:** Estimates of proportions of children younger than 5 years with a fever and *Plasmodium falciparum* infection who received an ACT in sub-Saharan countries in 2015

	Children <5 years with fever and infection who received an ACT
**Test positivity**	

RDT positive	197% (15.6–24.8)
RDT negative	16.3% (13.4–19.4)

**Residence**	

Rural	197% (15.4–25.4)
Urban	18.9% (9.9–31.3)

**Socioeconomic status**	

Poorer (<median wealth)	18.8% (14.4–24.8)
Wealthier (>median wealth)	21.2% (14.8–29.7)

**Endemicity**	

Hypoendemic	16.9% (8.3–30.6)
Mesoendemic	19.8% (15.9–24.6)
Hyperendemic or holoendemic	19.0% (9.9–35.3)

**Treatment seeking**	

Did seek care	25.9% (20.1–33.4)
Did not seek care	8.3% (5.2–13.0)

**Health-care provider**	

Private	18.7% (11.5–28.3)
Public	30.6% (22.8–41.2)

Estimates are mean (95% CI) and are for children younger than 5 years with a fever and an infection (RDT positive), apart from the test positivity data, which is for all children younger than 5 years with a fever. Data were calculated excluding those countries with a mean *Plasmodium falciparum* prevalence in children aged 2–10 years of less than 2%: Botswana, Djibouti, Eritrea, Ethiopia, South Africa, and Swaziland. Estimates were adjusted by the population at risk for each stratum. ACT=artemisinin-based combination therapy. RDT=rapid diagnostic test.

**Table 3:** Meta-analysis of factors associated with individual-level ACT coverage for children younger than 5 years with a fever and *Plasmodium falciparum* infection, in all children and those for whom treatment was sought

	All children with fever and positive RDT			All children with fever and positive RDT for whom treatment was sought		
	Number of surveys	Summary OR (95% CI)	p value	Number of surveys	Summary OR (95% CI)	p value
Age of child (>2 years *vs* ≤2 years)	73	1.09 (1.01–1.17)	0.0205	59	1.30 (1.20–1.40)	<0.0001
Caregiver's education (any *vs* none)	72	1.31 (1.22–1.41)	<0.0001	58	1.24 (1.13–1.36)	<0.0001
Household wealth (above *vs* below national median wealth index)	73	1.26 (1.16–1.39)	<0.0001	59	1.13 (0.99–1.27)	0.0530
Household insecticide-treated net ownership (yes *vs* no)	72	1.21 (1.13–1.29)	<0.0001	57	1.16 (1.07–1.27)	0.0005
Area of residence (urban *vs* rural)	70	1.18 (1.06–1.31)	0.0020	56	1.11 (0.97–1.27)	0.1329
*Pf*PR_2–10_	73	1.12 (1.02–1.23)	0.0193	59	1.20 (1.08–1.33)	0.0006
Health care (public *vs* private or other)	NA	NA	NA	54	3.18 (2.67–378)	<0.0001

Results of meta-analyses of individual survey-dataset-level regressions on ACT coverage for all children with fever and positive RDT results, and in the subgroup of those for whom treatment was sought. Surveys were excluded if fewer than five children who participated took an ACT. The number of surveys per parameter varied because for some surveys no variability was seen in that parameter. Multiple Indicator Cluster Surveys were excluded from the data of those who did seek treatment because they did not include information on where fever treatment was sought. RDT=rapid diagnostic test. ACT=artemisinin-based combination therapy. OR=odds ratio. *Pf*PR_2–10_=mean (logit-transformed) *P falciparum* prevalence in children aged 2–10 years. NA=not applicable. *Pf*PR_2–10_=mean (logit-transformed) *Plasmodium falciparum* prevalence in children aged 2–10 years.

## Research in context

### Evidence before this study

A wealth of nationally representative household survey data has become available over the past 10 years to help malaria programmes monitor and assess their progress in achieving high population coverage of effective interventions, including case management with effective diagnostic tools and treatment with antimalarials, which over the past decade have been artemisinin-based combination therapies (ACTs). Achieving a high rate of treatment with ACTs for all children with malaria is crucial to preventing severe disease and death. To gather evidence on progress in ACT treatment coverage before this study, we searched PubMed with the keywords “artemisinin combination therapy” and “malaria case management” in combination with “coverage”. We applied no language or publication date restrictions. The date of our last search was March 10, 2016. Several studies have used household survey data collected in single countries to examine ACT treatment rates for children with fever, and three recent studies have examined ACT treatment rates for children with fever for several countries across Africa using national household survey data. We found no studies that examined coverage of ACT treatment at the continental level over time for children with fever, or that examined ACT coverage for children with a *Plasmodium falciparum* infection from household survey data.

### Added value of this study

To our knowledge, this is the first study to estimate the coverage of ACT treatment for children younger than 5 years with fever and a P falciparum infection across sub-Saharan Africa over time.

### Implications of all the available evidence

Our results show that while coverage of ACT treatment has increased, it remains unacceptably low, and the likelihood of treatment is lower in rural, poorer populations and those who receive care through the private sector. Increased efforts are needed by malaria programmes and policy makers to ensure that treatments are available to patients who most need them, and inappropriate treatment of those without malaria infection is kept to a minimum.
