# Supplementary material for: Population coverage of artemisinin-based combination treatment in children younger than 5 years with fever and Plasmodium falciparum infection in Africa, 2003–2015: a modelling study using data from national surveys
Source: Lancet Glob Health. Author manuscript; Available in PMC 2018 Apr 1. (PMC5450656; doi:10.1016/S2214-109X(17)30076-1)
Supplement: supplement [file NIHMS860032-supplement-supplement.pdf]

THE LANCET
Global Health

Supplementary appendix

This appendix formed part of the original submission and has been peer reviewed. We post it as supplied by the authors.

Supplement to: Bennett A, Bisanzio D, Yukich JO, et al. Population coverage of artemisinin-based combination treatment in children younger than 5 years with fever and Plasmodium falciparum infection in Africa, 2003–2015: a modelling study using data from national surveys. *Lancet Glob Health* 2017; **5:** e418–27.

Supplementary Material

Data on children with fever and *P.falciparum* infection treated with an ACT

The full dataset included 833,419 children <5 years old with fever status from 22 MIS surveys, 61 DHS surveys, and 20 MICS surveys for 33 countries in sub-Saharan Africa from 2003-2015.

RDT data were collected for 145,529 children under 5 from 40 surveys (19 DHS, 20 MIS, 1 MICS). Among these children, 144,130 had fever status in the past two weeks recorded, with 40,261 reporting fever in the past two weeks (27·9%). Across these children with fever status and RDT collected, the RDT positivity rate was almost two times higher in those reporting fever in the past two weeks (39·2%) than in those without fever (22·1%). A comparison of the RDT positivity rate for children with fever and those without fever is shown by survey in Figure 1.

Figure 1. Comparison of survey-weighted RDT prevalence for children with and without reported fever in the past two weeks, for surveys with RDT data collected. Labels represent concatenated country code, year, and survey type (DHS=0, MIS=1, MICS=2).

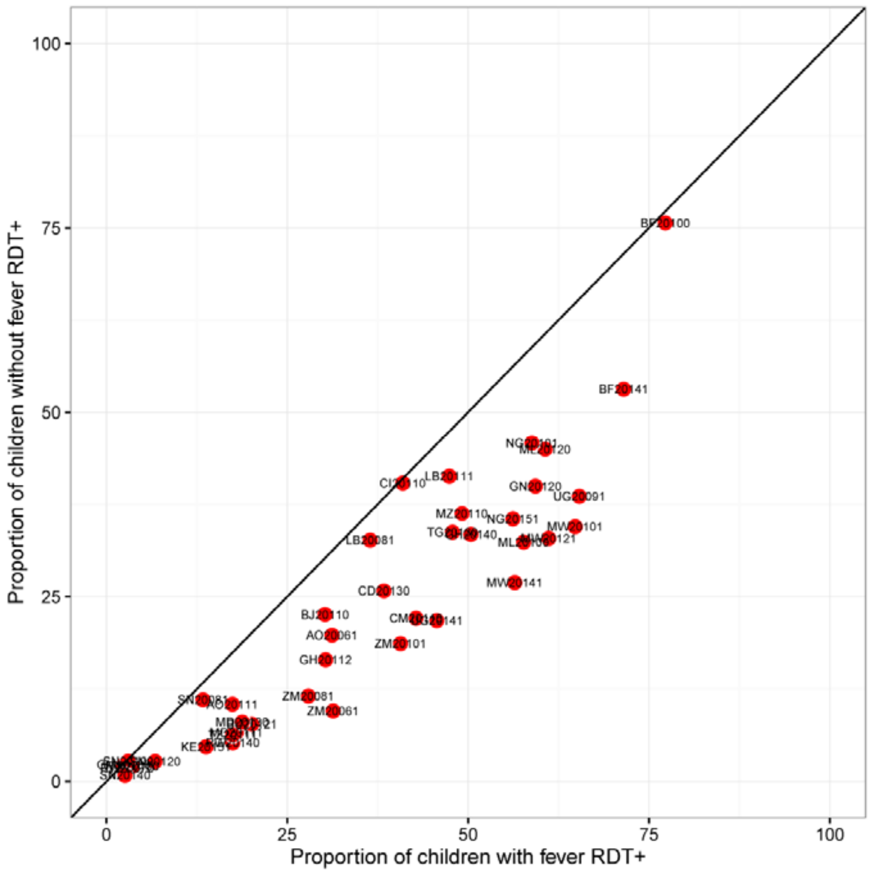

Among children with fever in the past two weeks and RDT status recorded, 39,121 had information on treatment seeking recorded. Of these, 25,007 (63·9%) reported seeking some form of treatment for their fever. RDT positivity

rates are shown by survey for those who sought treatment and those who did not in Figure 2; this plot demonstrates that for the majority of surveys, children who did not seek care were more likely to have a positive RDT.

Figure 2. Comparison of survey-weighted RDT prevalence for children with reported fever in the past two weeks who sought treatment versus those who did not seek treatment, for surveys with RDT data collected. Labels represent concatenated country code, year, and survey type (DHS=0, MIS=1, MICS=2).

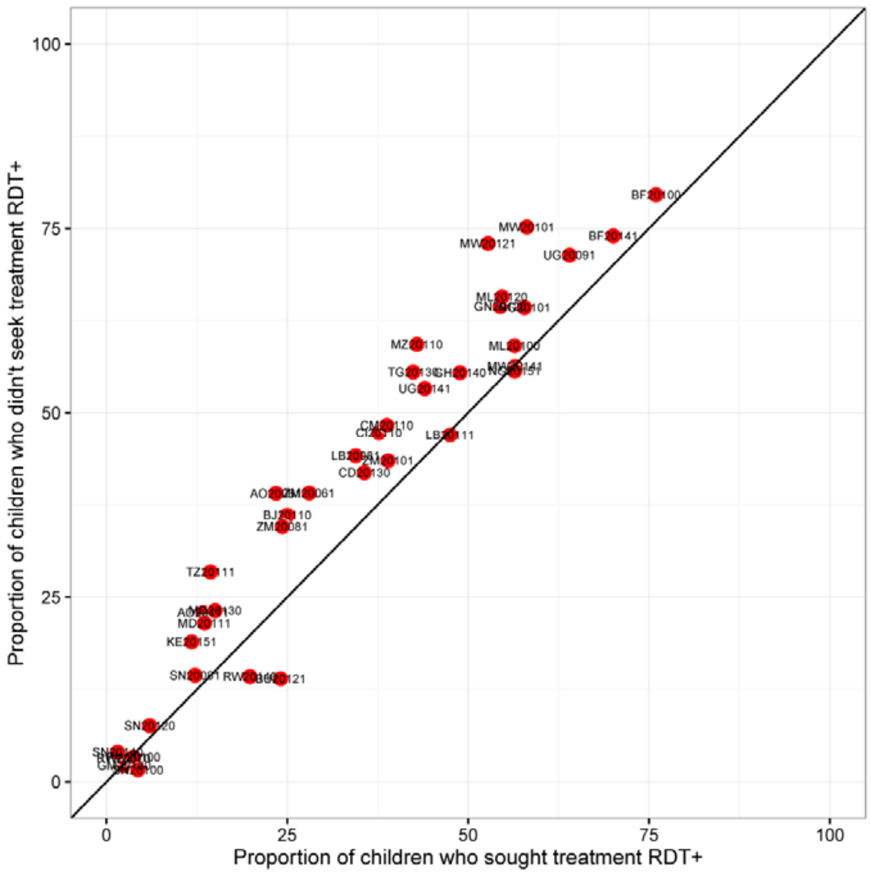

A sensitivity analysis was conducted for those surveys that collected data on the time since the start of a fever episode. This included 12 MIS surveys and 1 DHS survey. For these surveys, we compared estimates of ACT coverage for RDT+ children for the reference period (fever in the past two weeks) and including only those children reporting fever within the past 2-7 days. We found only very slight, non-systematic differences between the estimates for each country (Figure 3).

Figure 3. Comparison of the proportion of RDT+ children receiving an ACT for fever in the past 2 weeks, versus fever in the past 1 week, for surveys with these data available. Labels represent concatenated country code, year, and survey type (DHS=0, MIS=1, MICS=2).

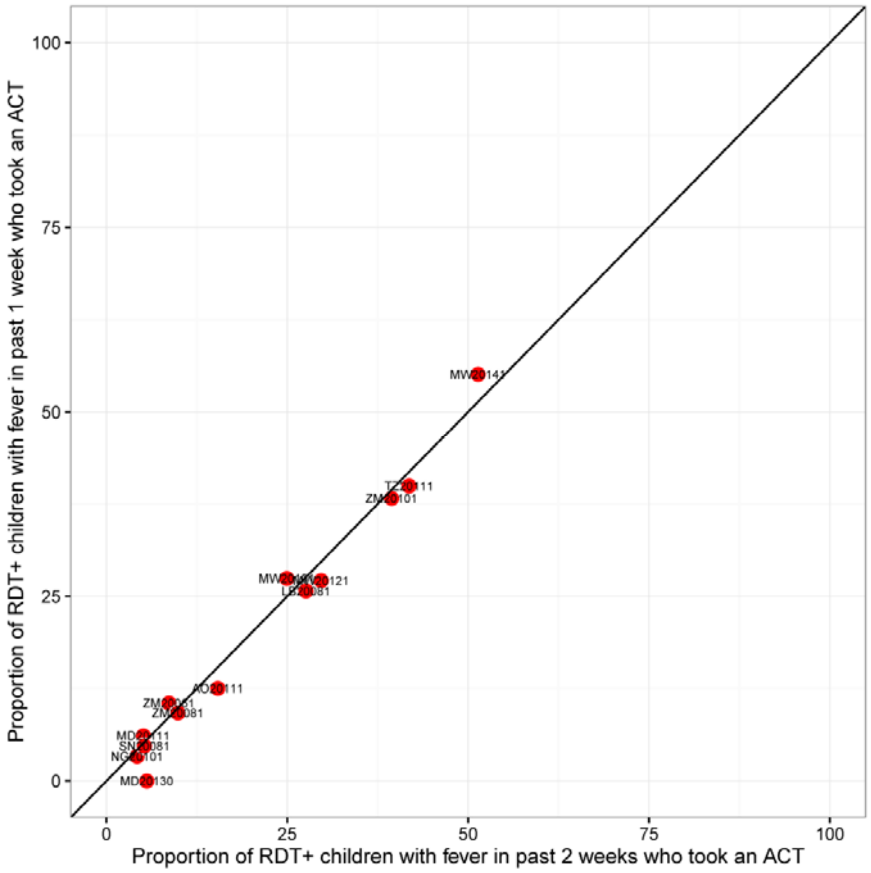

Model procedures for children without RDT collected

A detailed sampling procedure was undertaken to predict RDT status for children without RDT collected, and to propagate the uncertainty in these predictions through the modeling procedure (Figure 4).

Annual estimates of *Pf*PR in children 2-10 years old (*Pf*$PR_{2\text{-}10}$) at high spatial resolution (1km x 1km) were obtained from the Malaria Atlas Project (MAP) [1]. *Pf*$PR_{2\text{-}10}$ estimates were extracted at the sampled cluster level and year of survey for household surveys with geographic coordinates. For surveys without geographic coordinates, we extracted the median of the second administrative level (usually district) for each cluster and year of the survey.

We used all available survey datasets with RDT results at the time of survey to create a logistic regression model to predict malaria parasite infection amongst febrile children. We assumed that a positive RDT provides a reasonable measure of a 2 week period prevalence of infection as it detects the parasite antigen that most often persist up to 2 weeks after an infection has been cleared, which is supported by previous research and RDT evaluations [2-4]. We included the child's age and sex, household wealth quintile and ITN ownership, urban/rural status, season (rainy/dry) and malaria transmission intensity as measured by *Pf*$PR_{2\text{-}10}$ in the regression model predicting RDT status. RDT status was predicted for the remaining 161,443 children (80%). In the RDT prediction model, increasing age, decreasing wealth, lack of household ITN ownership, increasing cluster *Pf*$PR_{2\text{-}10}$, rural location, and survey conducted during the rainy season were all strongly associated with RDT positive propensity. This model achieved good predictive accuracy, with a mean area under the curve (AUC) of 0·78 from 100 independent 15% samples.

To account for uncertainty and obtain predictions of RDT status among all children with a fever from the compiled surveys, we sampled 100 values from the posterior distribution of PfPR$_{2\text{-}10}$ at each survey location and time, and used the coefficients estimated from each of 100 logistic model estimations to produce 100 separate predicted probabilities of RDT status for each child. We then sampled 10 (1,0) values using the binomial distribution for each of the 100 predicted probabilities, to produce a total of 1,000 predictions for each child. For each of these child-level predictions we then calculated the national survey-weighted proportion of children <5 with a fever and positive RDT test (as measured, or predicted if not measured) and whether they received an ACT for all surveys. A graphic of this process is summarized in Figure 4.

Figure 4. Flow of sampling and modeling process.

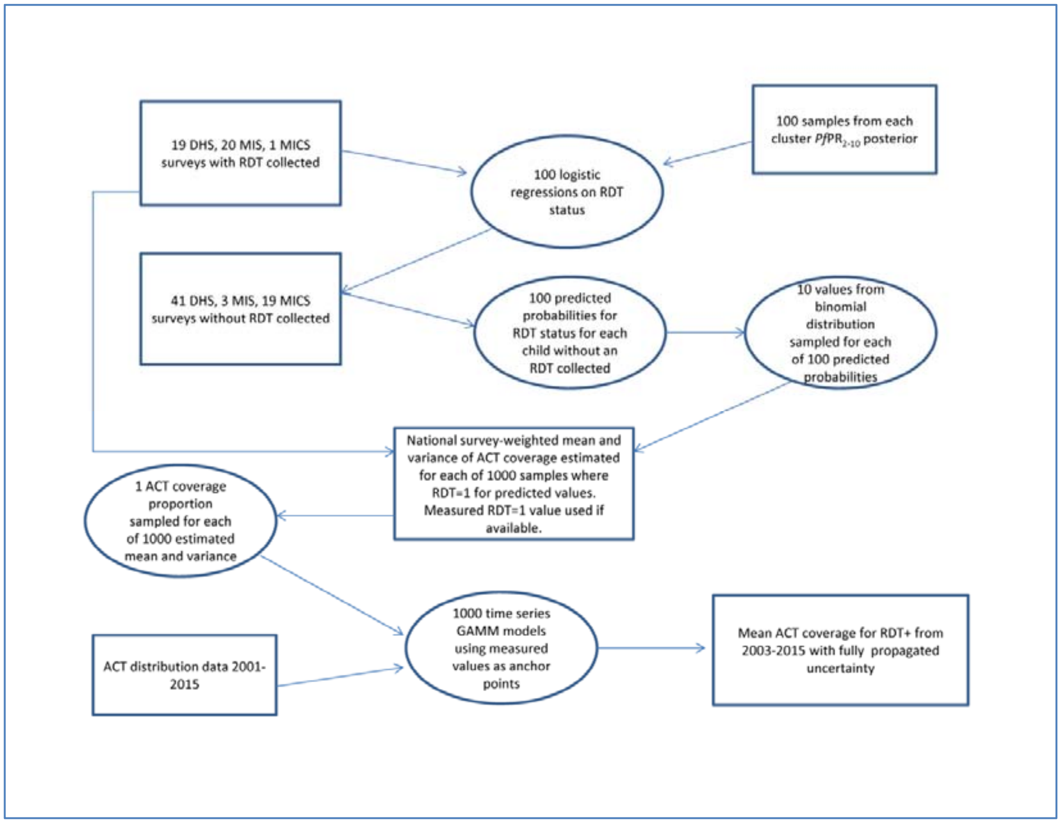

Figure 5. Proportion of RDT+ vs RDT- children receiving an ACT for fever, by country weighted by population at risk. Panel A includes only those surveys and observations with RDT data collected. Panel B includes surveys without RDT data collected, where RDT data were predicted. Labels represent concatenated country code, year, and survey type (DHS=0, MIS=1, MICS=2).

A. Surveys with measured RDT

B. Surveys for which RDT result predicted

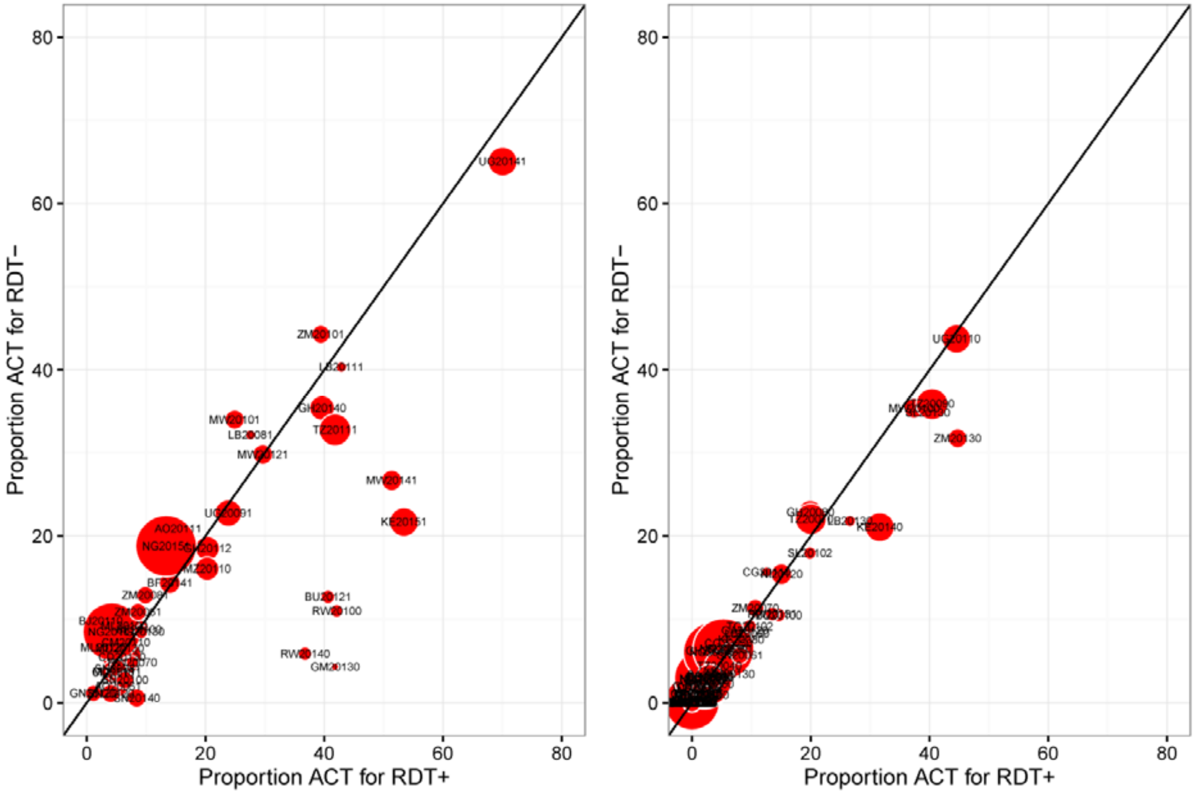

Figure 6. Proportion of RDT+ children with fever receiving ACT for surveys where RDT status was collected, compared to the ACT coverage estimates from the same surveys with RDT+ predicted from the other available surveys.

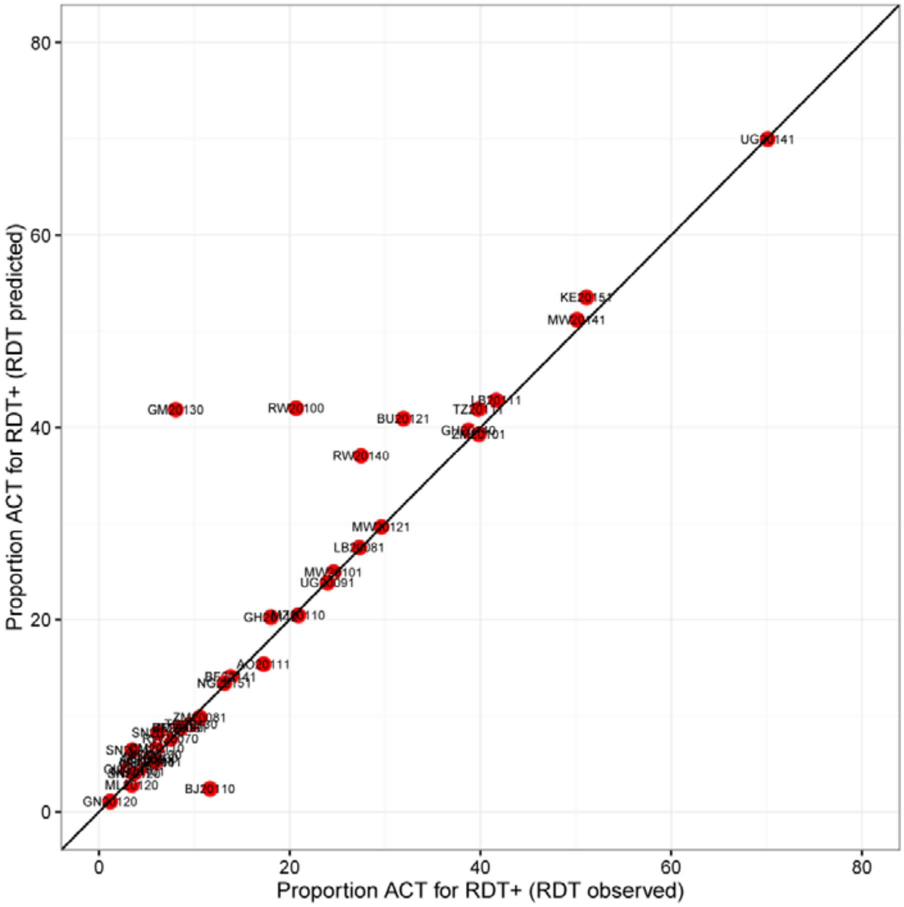

Table 1. Survey-specific validation statistics (area under the curve (AUC)) for the RDT prediction model, where each survey's value represents the comparison of observed RDT status and predicted values when that survey was held out of model fitting.

Survey	AUC (95% CI)	Survey	AUC (95% CI)
AO20061	0.82 (0.80-0.84)	MW20121	0.72 (0.70-0.74)
AO20111	0.84 (0.81-0.87)	MW20141	0.71 (0.66-0.75)
BF20100	0.65 (0.64-0.67)	MZ20110	0.77 (0.76-0.79)
BF20141	0.61 (0.56-0.65)	NG20101	0.62 (0.59-0.65)
BJ20110	0.72 (0.69-0.73)	NG20151	0.65 (0.59-0.69)
BU20121	0.74 (0.73-0.76)	RW20070	0.73 (0.69-0.76)
CD20130	0.71 (0.70-0.72)	RW20100	0.77 (0.74-0.79)
CI20110	0.66 (0.63-0.70)	RW20140	0.73 (0.68-0.76)
CM20110	0.72 (0.70-0.74)	SN20081	0.68 (0.65-0.71)
GH20112	0.59 (0.58-0.59)	SN20100	0.70 (0.68-0.73)
GH20140	0.62 (0.59-0.68)	SN20120	0.74 (0.64-0.81)
GM20130	0.72 (0.63-0.80)	SN20140	0.86 (0.82-0.88)
GN20120	0.73 (0.72-0.74)	TG20130	0.65 (0.59-0.72)
KE20151	0.79 (0.68-0.85)	TZ20111	0.80 (0.77-0.82)
LB20081	0.63 (0.60-0.66)	UG20091	0.74 (0.72-0.75)
LB20111	0.70 (0.69-0.72)	UG20141	0.61 (0.56-0.66)
MD20111	0.81 (0.79-0.83)	ZM20061	0.77 (0.71-0.80)
MD20130	0.84 (0.82-0.86)	ZM20081	0.80 (0.78-0.82)
ML20120	0.81 (0.79-0.82)	ZM20101	0.83 (0.82-0.84)
MW20101	0.76 (0.75-0.78)		

Table 2. For each survey included, the proportion of children <5 with fever taking any antimalarial, the proportion of children <5 with fever and *Pf* taking any antimalarial, and the proportion of children <5 with fever and *Pf* taking an ACT.

Survey	Proportion of children<5 with fever taking any antimalarial	Proportion of children <5 with fever and *Pf* taking any antimalarial	Proportion of children <5 with fever and *Pf* taking an ACT
AO20061	28.0 (20.3-35.6)	21.0 (10.0-35.7)	4.3 (0.8-12.4)
AO20111	28.4 (25.1-32.0)	23.7 (16.8-31.9)	17.0 (11.3-24.4)
BF20030	49.5 (46.2-53.0)	49.5 (45.3-53.3)	0.0 (0.0-0.0)
BF20062	46.3 (40.3-52.0)	44.4 (37.8-52.3)	0.0 (0.0-0.1)
BF20100	35.1 (32.8-37.5)	34.1 (31.3-37.2)	8.2 (6.8-9.7)
BF20141	49.3 (45.9-52.7)	50.2 (46.5-54.2)	13.7 (11.4-16.4)
BJ20060	54.1 (51.7-56.5)	53.4 (50.4-56.7)	0.6 (0.3-1.1)
BJ20110	38.2 (35.2-41.7)	32.7 (26.4-39.2)	11.5 (7.6-16.0)
BU20062	6.8 (5.4-8.4)	7.1 (4.7-10.4)	3.1 (1.6-5.2)
BU20100	17.3 (14.8-19.7)	20.1 (15.8-24.7)	14.5 (10.5-19.0)
BU20121	25.4 (22.1-28.8)	42.1 (36.1-49.0)	31.9 (26.3-38.1)
CA20062	39.5 (36.3-42.7)	40.2 (35.7-44.6)	2.3 (0.9-4.3)
CA20102	31.9 (28.8-35.0)	32.0 (27.5-36.4)	2.9 (1.6-5.0)
CD20070	29.8 (26.4-33.9)	28.6 (23.9-33.6)	0.4 (0.1-0.9)
CD20102	39.6 (36.3-43.1)	38.6 (34.2-43.2)	1.4 (0.6-2.5)
CD20130	29.2 (27.0-31.9)	29.5 (25.4-34.0)	5.7 (4.1-7.9)
CG20050	47.9 (43.6-51.9)	49.0 (41.6-56.4)	5.8 (2.6-11.2)
CG20110	25.0 (21.4-28.8)	23.6 (17.0-30.2)	12.4 (7.1-19.0)
CI20062	36.0 (32.7-39.3)	34.3 (29.8-38.6)	2.6 (1.4-4.3)
CI20110	17.5 (14.8-20.6)	16.7 (13.1-21.4)	2.9 (1.5-5.2)
CM20040	66.5 (63.2-69.5)	66.6 (61.9-71.0)	0.0 (0.0-0.0)
CM20110	23.2 (20.5-26.1)	25.9 (21.9-30.0)	6.0 (4.1-8.2)
GA20120	25.7 (21.0-31.3)	26.3 (15.1-39.2)	8.4 (3.0-17.7)
GH20030	62.8 (58.8-66.9)	62.3 (55.7-68.4)	0.0 (0.0-0.0)
GH20062	61.0 (55.6-66.2)	60.2 (52.4-67.2)	2.7 (1.0-5.9)
GH20080	43.0 (36.9-48.5)	41.9 (33.5-50.6)	19.8 (13.5-27.7)
GH20112	52.6 (47.8-57.3)	57.8 (50.6-64.1)	17.9 (14.1-22.1)
GH20140	48.4 (43.0-53.6)	48.9 (41.4-55.6)	38.8 (31.7-45.2)
GM20062	62.7 (58.1-67.4)	64.5 (50.7-77.9)	0.0 (0.0-1.1)
GM20130	6.6 (4.6-8.8)	9.5 (1.6-25.5)	7.3 (1.0-20.2)
GN20050	43.4 (39.7-46.9)	43.3 (38.6-48.4)	0.4 (0.0-1.2)
GN20120	28.0 (24.9-31.4)	27.1 (22.6-31.3)	1.1 (0.5-2.1)
GW20062	45.7 (41.3-50.2)	41.8 (30.2-55.0)	0.5 (0.0-4.1)
KE20030	26.2 (23.5-29.0)	34.0 (29.0-39.2)	0.0 (0.0-0.0)
KE20080	23.0 (19.6-26.6)	28.8 (20.3-38.7)	8.0 (3.5-14.3)
KE20140	27.0 (24.8-29.3)	35.5 (27.2-43.0)	31.7 (23.7-39.0)
KE20151	26.7 (21.7-32.7)	53.3 (42.5-65.0)	51.2 (41.0-61.8)
LB20060	58.9 (54.3-63.1)	59.2 (52.9-64.9)	9.1 (5.9-13.0)
LB20081	66.8 (62.7-70.9)	65.2 (59.1-71.3)	27.3 (20.9-33.3)
LB20111	56.9 (53.1-60.4)	56.7 (52.1-60.7)	41.6 (36.1-47.1)
LB20130	55.8 (52.4-59.1)	57.0 (51.6-61.7)	26.4 (21.4-32.0)

MD20030	34.2 (28.9-39.8)	35.6 (26.7-44.4)	0.0 (0.0-0.0)
MD20080	19.6 (16.7-22.9)	21.6 (14.7-30.1)	0.7 (0.0-3.2)
MD20111	19.9 (15.5-25.1)	31.4 (21.1-43.6)	4.8 (2.0-9.9)
MD20130	11.2 (7.7-15.3)	12.9 (5.9-23.6)	5.1 (1.1-12.0)
ML20060	48.1 (44.7-52.0)	48.4 (43.0-53.6)	0.0 (0.0-0.0)
ML20100	34.1 (27.9-40.3)	37.5 (28.8-46.4)	6.3 (2.6-11.7)
ML20120	22.5 (18.9-26.8)	20.5 (15.3-26.9)	3.4 (1.5-5.9)
MR20072	12.1 (6.6-18.7)	12.9 (0.0-44.5)	0.0 (0.0-0.0)
MW20040	58.1 (55.6-60.6)	59.1 (55.0-63.1)	0.0 (0.0-0.0)
MW20062	24.9 (23.1-26.8)	24.6 (21.7-27.5)	0.2 (0.0-0.5)
MW20100	43.4 (41.4-45.3)	43.8 (41.0-46.8)	37.4 (34.6-40.2)
MW20101	31.0 (26.8-35.2)	27.2 (22.5-32.2)	24.6 (19.8-29.8)
MW20121	32.3 (28.1-37.0)	32.6 (27.8-37.8)	29.6 (25.1-34.4)
MW20141	42.5 (36.5-48.6)	53.1 (44.3-61.2)	50.3 (41.6-58.7)
MZ20030	14.9 (12.7-17.2)	15.5 (12.5-18.9)	0.0 (0.0-0.0)
MZ20082	26.5 (23.3-29.5)	28.1 (23.3-32.9)	0.9 (0.2-2.3)
MZ20110	30.1 (26.7-34.2)	34.0 (28.5-40.2)	20.7 (15.4-27.1)
NG20030	34.2 (30.9-37.6)	33.9 (28.7-39.3)	0.0 (0.0-0.0)
NG20072	52.0 (48.7-54.9)	50.4 (45.2-55.1)	2.0 (1.0-3.4)
NG20080	33.1 (30.5-35.6)	31.5 (28.0-34.9)	1.9 (1.2-2.7)
NG20101	49.0 (44.4-53.4)	46.7 (41.0-52.1)	4.0 (2.7-5.8)
NG20112	44.6 (42.0-47.1)	41.5 (37.7-45.2)	3.6 (2.4-5.1)
NG20130	32.7 (30.1-35.3)	31.0 (26.8-35.2)	5.3 (3.6-7.2)
NG20151	41.3 (37.5-44.7)	38.8 (35.1-43.1)	13.1 (10.8-16.0)
NI20060	33.1 (29.6-36.7)	32.8 (26.5-39.4)	0.0 (0.0-0.0)
NI20120	19.1 (16.5-22.1)	19.0 (14.0-24.4)	15.0 (10.6-20.1)
NM20060	9.8 (7.6-12.3)	11.6 (5.2-19.5)	2.6 (0.2-7.9)
NM20130	8.4 (6.0-11.1)	9.3 (2.4-18.6)	5.8 (1.0-15.5)
RW20050	15.3 (13.4-17.6)	17.6 (12.3-23.7)	0.0 (0.0-0.0)
RW20070	5.6 (4.2-7.3)	6.9 (1.0-21.9)	6.5 (0.8-19.7)
RW20100	10.8 (8.8-13.0)	21.0 (11.5-33.9)	20.3 (11.1-33.3)
RW20131	11.9 (9.3-15.1)	14.0 (5.7-26.0)	13.1 (4.8-24.7)
RW20140	11.2 (9.1-13.6)	27.6 (20.2-36.2)	27.4 (18.9-35.8)
SL20052	51.9 (48.8-55.1)	51.8 (47.4-56.6)	1.1 (0.4-2.2)
SL20080	30.0 (25.4-34.5)	29.1 (23.2-35.9)	6.1 (3.3-9.8)
SL20102	62.1 (59.3-64.9)	63.0 (59.5-66.5)	19.8 (16.6-23.5)
SL20130	48.2 (45.0-51.4)	50.8 (45.5-56.1)	39.7 (34.2-45.1)
SN20050	26.7 (23.4-30.2)	23.0 (18.3-28.4)	0.0 (0.0-0.0)
SN20061	19.9 (17.1-23.2)	22.6 (16.7-29.5)	7.9 (4.5-12.6)
SN20081	9.1 (7.5-10.9)	9.5 (6.5-13.5)	5.7 (3.6-9.1)
SN20100	8.1 (6.2-10.3)	7.6 (3.5-14.4)	3.1 (1.0-8.1)
SN20120	6.1 (3.9-8.9)	8.7 (3.9-16.8)	3.4 (1.0-7.8)
SN20140	6.5 (3.2-10.9)	5.2 (1.1-15.3)	5.3 (1.1-16.1)
SO20062	7.9 (6.1-10.0)	7.7 (4.3-12.1)	0.6 (0.0-2.7)
TG20062	47.9 (42.8-52.9)	46.8 (40.0-53.7)	1.0 (0.2-2.6)

TG20102	36.5 (33.3-39.8)	36.1 (32.3-40.2)	9.7 (7.4-12.3)
TG20130	18.3 (15.4-21.4)	19.1 (15.2-24.0)	9.5 (7.0-12.8)
TZ20040	58.3 (54.4-61.9)	59.3 (53.7-65.2)	4.6 (2.5-7.3)
TZ20070	56.6 (51.9-61.5)	55.3 (47.4-62.8)	20.0 (14.6-25.7)
TZ20090	59.1 (55.4-62.5)	61.5 (53.9-68.0)	40.5 (33.0-47.8)
TZ20111	53.6 (50.4-57.1)	58.0 (50.5-66.0)	39.9 (32.2-46.8)
UG20060	61.3 (58.9-63.9)	62.1 (58.3-65.3)	3.0 (1.8-4.4)
UG20091	59.9 (54.7-64.5)	63.4 (57.4-68.9)	23.8 (19.0-29.5)
UG20110	64.5 (61.3-67.8)	65.1 (60.6-69.6)	44.6 (39.8-49.4)
UG20141	77.3 (73.9-79.9)	80.2 (75.8-83.8)	70.1 (65.6-74.5)
ZM20061	50.2 (42.4-58.3)	38.8 (30.1-47.7)	8.2 (3.3-15.7)
ZM20070	38.5 (34.4-42.1)	37.8 (30.3-45.6)	10.4 (5.8-16.6)
ZM20081	46.7 (41.9-51.5)	41.5 (34.4-48.7)	10.3 (6.1-16.2)
ZM20101	55.5 (51.1-59.7)	57.0 (50.0-63.7)	39.7 (34.2-45.8)
ZM20130	39.8 (36.4-43.0)	48.3 (42.8-54.4)	44.7 (39.3-50.4)
ZW20050	4.6 (2.7-7.2)	6.3 (0.4-19.8)	0.0 (0.0-0.0)
ZW20100	2.2 (0.9-4.2)	2.7 (0.0-12.6)	1.3 (0.0-9.3)

Data on number of ACT distributed
We acquired National Malarial Control Program (NMCP) annual country level ACT distribution data for 2001 to 2014. ACT distributions reported by country programs represent those going to the public sector, which accounts for more than two-thirds of total global ACT sales. The number of ACTs distributed through the private sector in each country is not available. However, a similar trend is observed in global public and private sector ACT sales [5], and therefore NMCP ACT distributions are a reasonable approximation of ACT availability in each country.

For 2015, ACT distributions were estimated by computing the mean of ACTs distributed from 2012 to 2014. ACT distribution data were available for 65% of country – year time points (Figure 7), and ACT availability per capita showed a significant and strong correlation with ACT coverage (Sperman's rho = 0·72, p<0·001). Gap filling procedures for missing ACT distribution and ACT coverage data points are described below.

Figure 7. Time series of ACT distributed per each country acquired from National Malaria Control Programs via the World Malaria Report.

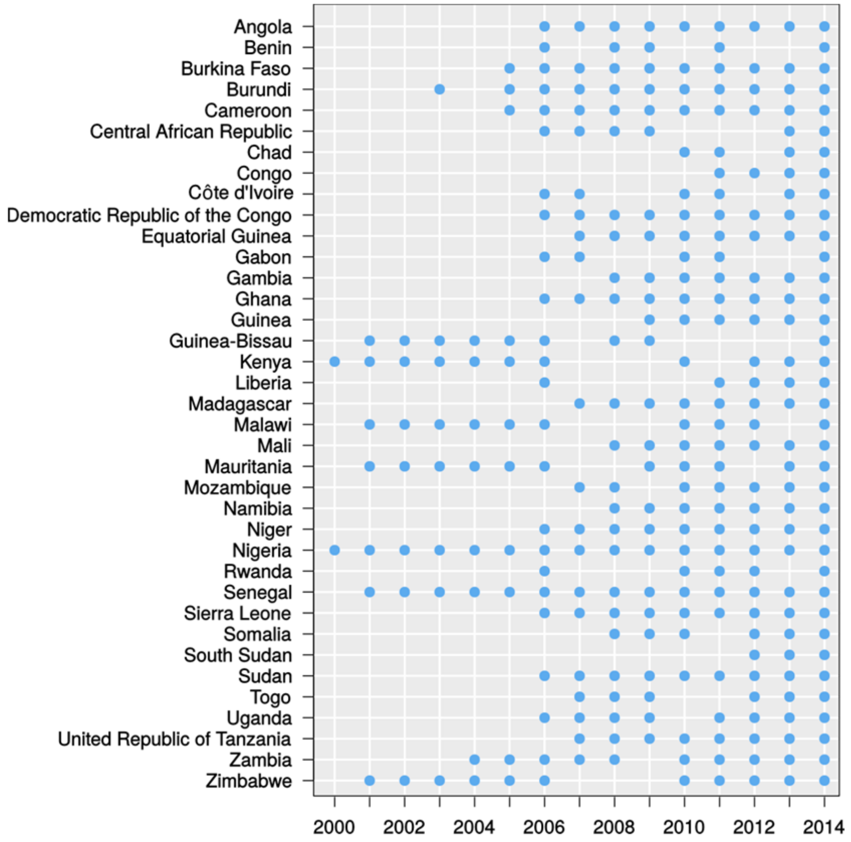

ACT distribution data were standardized to ACT per capita "availability" (ACT_{cap}), where the annual country level ACT per capita is the number of ACT distributed divided by the population at risk for each country and year. The population at risk for each country was obtained by extracting data from interpolations of the five yearly WorldPop [6] multiplied by the proportion of the population at either high or low risk from the World Malaria Report 2013 [7].

Statistical model

We imputed ACT coverage values for each country and year (2003-2015) with no survey dataset available using a generalized additive mixed-model (GAMM) that took into account the spatial and temporal sparseness of the data and incorporated the relationship between ACT coverage and ACT distribution data across countries. National annual ACT coverage was modelled as a function of time, country, AFRO region, and ACT_{cap}. This final full model was parameterized as follows:

$$ACT_{rdt+} \sim f(Year)+ +f(ACT_{cap-3y})+f_{rand}(Country)+f_{rand}(Region)$$

Where ACT_{rdt+} is the country-level proportion of children <5 with a fever plus a malaria parasite infection that received an ACT, $f(Year)$ is a non-linear effect of time in years, and $f(ACT_{cap-3y})$ is a non-linear function of the mean of ACT_{cap} distributed during the given year and previous two years, $f_{rand}(Country)$ is a country-level random effect and $f_{rand}(Region)$ is an AFRO region random effect. Gaps in the ACT coverage time series for each country were filled by predicting estimates based on posterior means of the model's fixed effect, using known values as anchor points. A separate GAMM model including year, country, and AFRO region was used to fill gaps in the time series of ACT_{cap}.

Gap filling procedure

In our filling procedure, we used known data points to train the model as anchor points to estimate the missing values in each country time series. Because the covariates in our model did not describe all the factors affecting ACT coverage in a country (ie. health system efficiency, health care seeking, ACT distribution chain), we assume that the known point could be used to represent the overall effect of many variables not included in the model. Thus, we applied the GAMM results with anchor points to calculate the missing values. We applied the following two formulas to estimate the missing data of ACT per capita and ACT coverage time series, respectively:

$$ACT_{cap},_{k} = exp(log(ACT_{cap,k-1}) - f(year)_{k-1} + f(year)_{k}) \quad \text{(eq. 1)}$$
$$ACT_{cap},_{k} = exp(log(ACT_{cap,k+1}) - f(year)_{k+1} + f(year)_{k}) \quad \text{(eq. 2)}$$
$$ACT_{frdt+},_{k} = exp(log(ACT_{rdt+,k-1}) - f(ACT_{cap})_{k-1} + f(ACT_{cap})_{k}) \quad \text{(eq. 3)}$$
$$ACT_{rdt+},_{k} = exp(log(ACT_{rdt+,k+1}) - f(ACT_{cap})_{k+1} + f(ACT_{cap})_{k}) \quad \text{(eq. 4)}$$

Where *k* represent the year with missing data, *k-1* the year before the missing value, *k+1* the year after the missing value, ACT_{cap} is ACT per capita, ACT_{rdt+} is the percentage of children with fever and a *Pf* infection treated with an ACT, *f(year)* and $f(ACT_{cap})$ are the effect of the year and ACT_{cap} as non-linear function from GAMM, respectively. When the missing values were between two anchor points we applied *eq.1* and *eq.2* to calculate estimates year by year. The final value for each year was calculated as the mean of the estimates of the two equations. Per each randomization, values of *f(year)* and $f(ACT_{cap})$ were sampled from the posterior distribution of GAMMs.

Model validation

The performance of our forecasting methods was evaluated using the root mean square error (RMSE), mean absolute error (MAE), and the mean absolute scaled error (MASE). When MASE<1, the model has better prediction power than näive forecasting. In the näive technique the forecasts are simply set to be equal to the value of the last observation. We calculated prediction accuracy metrics using randomizations with 70% of data to train the model and 30% to test the model prediction. The data used to test the model were not used as anchor points during prediction. All indices were calculated for each model realization. The model showed RMSE=7·4 (range: 5·2-11.2), MAE=6·4 (range: 4·6-9·2), and MASE=0·79 (range: 0·65-0·9). Figure 8 shows a scatter plot of observed and estimated values.

Figure 8. Scatter plot of comparison between values of ACT coverage dropped from the dataset and predicted estimates. Values and estimates of ACT coverage were the mean of all randomizations.

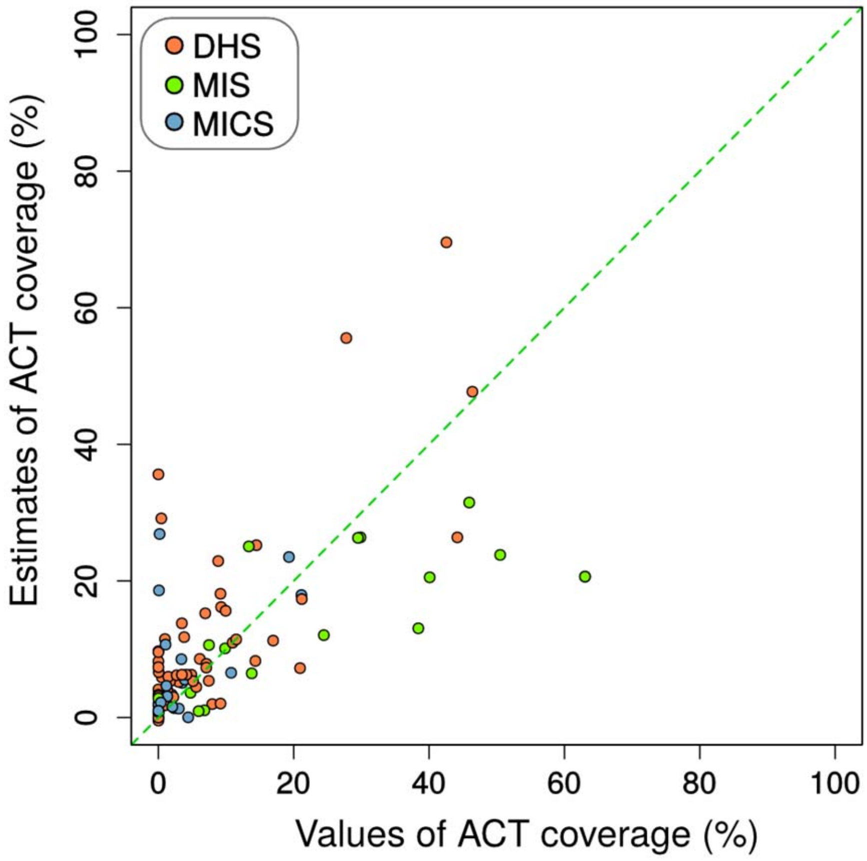

Comparing the predicted ACT availability per capita in 2015 ($ACT_{cap\text{-}3y} \times 100$) with predicted ACT coverage, we found that 9 (24·3%) countries showed a gap of at least 10% between the two values (Figure 9).

Figure 9. Difference between predicted availability of ACTs and the predicted percent of children with fever + *P. falciparum* receiving an ACT, by country for 2015. The predicted availability of ACTs per capita in 2015 for fever + *P. falciparum* of each country was calculated as $ACT_{cap\text{-}3y} \times 100$.

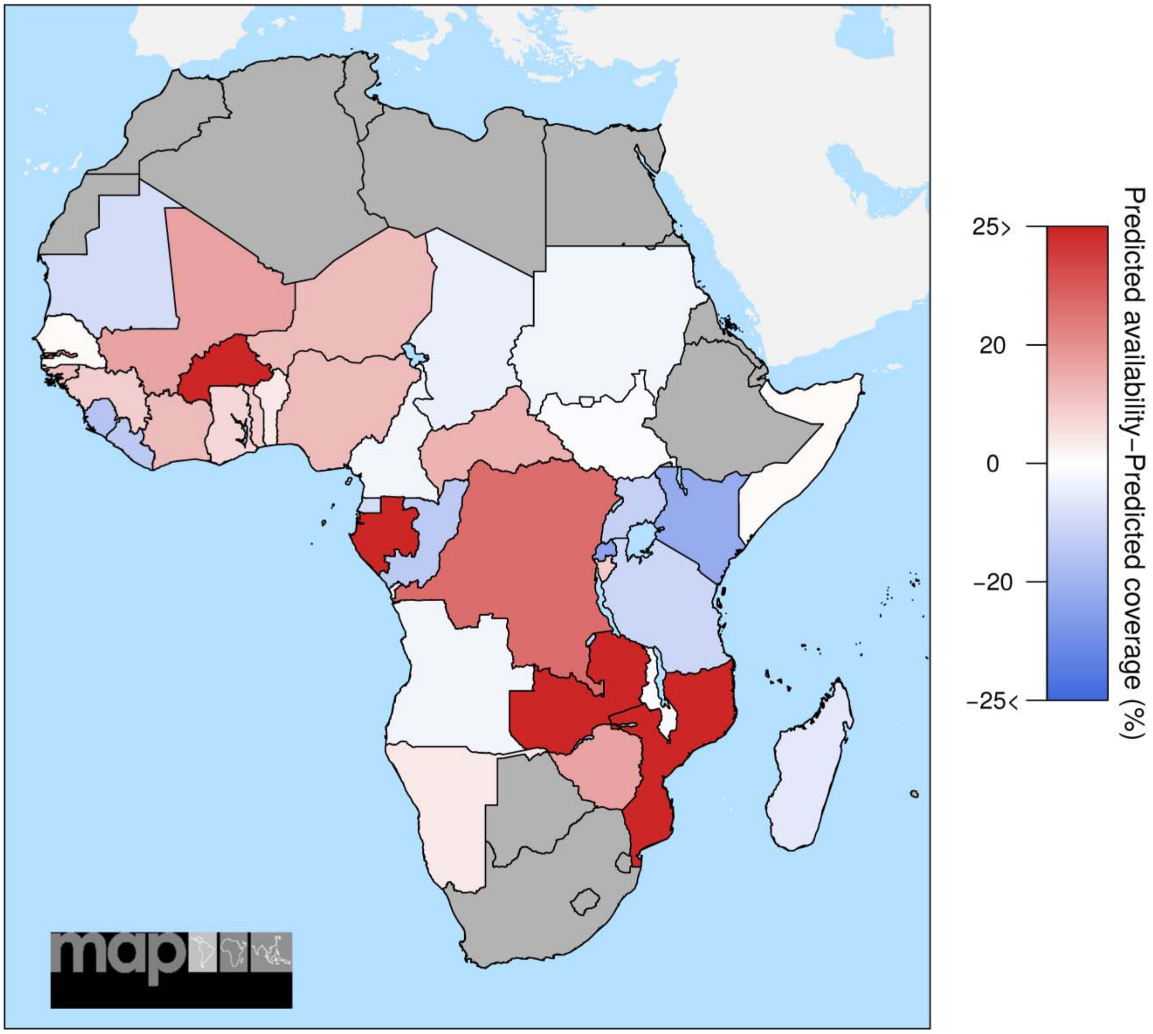

Table 3 below includes the predicted ACT coverage for children<5 with fever and P. falciparum infection in 2005, 2010, and 2015, and Figure 10 that follows includes the complete time series for each country from 2000-2015.

Table 3. Final predicted ACT coverage for children<5 with fever and *P. falciparum* infection by country in 2005, 2010, and 2015.

**Country**	**2005 (95% CI)**	**2010 (95% CI)**	**2015 (95% CI)**
Angola	1.7 (0.3-5.5)	12.4 (8.6-18.7)	14.1 (9.1-20.7)
Benin	0.3 (0.1-0.6)	7.2 (5.0-9.7)	7.2 (4.7-10.6)
Botswana	3.1 (2.1-5.0)	5.6 (3.9-8.7)	8.5 (5.7-13.6)
Burkina Faso	0.0	5.7 (4.9-6.8)	14.0 (11.4-16.5)
Burundi	1.4 (0.8-2.4)	12.0 (8.9-15.6)	45.2 (35.6-54.7)
Cameroon	1.0 (0.7-1.3)	4.7 (3.2-6.3)	5.6 (3.8-7.6)
Central African Republic	1.1 (0.5-2.1)	4.0 (2.6-5.7)	1.6 (0.7-2.9)
Chad	2.7 (1.8-4.5)	5.6 (3.8-8.7)	9.9 (6.9-15.4)
Democratic Republic of Congo	0.1 (0.0-0.4)	1.1 (0.5-1.9)	6.1 (4.4-8.5)
Djibouti	3.3 (2.2-5.2)	6.9 (4.7-10.3)	10.0 (6.8-15.4)
Equatorial Guinea	3.1 (2.1-4.9)	6.9 (4.9-10.3)	10.6 (7.4-16.4)
Eritrea	2.7 (1.8-4.5)	4.9 (3.3-7.8)	8.9 (6.0-14.2)
Ethiopia	3.2 (2.1-5.1)	9.0 (6.4-13.1)	14.0 (9.8-20.6)
Gabon	0.8 (0.3-1.7)	5.7 (2.6-11.5)	13.0 (4.8-29.1)
Gambia	0.0 (0.0-0.1)	8.4 (2.6-20.6)	5.7 (1.0-15.7)
Ghana	1.0 (0.3-2.5)	17.8 (13.8-22.1)	43.9 (36.1-51.7)
Guinea	0.3 (0.0-0.9)	1.8 (1.1-2.8)	1.2 (0.6-2.1)
Guinea-Bissau	0.2 (0.0-1.8)	2.6 (0.5-9.3)	2.6 (0.5-16.8)
Côte d’Ivoire	1.2 (0.7-2.0)	2.8 (1.7-4.1)	3.3 (1.6-5.8)
Kenya	0.7 (0.2-1.7)	24.9 (18.1-34.4)	41.2 (34.9-47.3)
Liberia	3.1 (2.0-4.4)	38.1 (32.8-44.3)	12.4 (9.4-16.6)
Madagascar	0.2 (0.0-1.0)	3.0 (1.4-5.7)	5.0 (1.1-11.5)
Malawi	0.0 (0.0-0.1)	48.5 (44.0-53.6)	51.8 (43.0-60.3)
Mali	0.0	5.9 (2.4-10.7)	4.3 (2.1-7.0)
Mauritania	0.0	4.3 (2.9-6.7)	8.7 (6.0-13.8)
Mozambique	0.0 (0.0-0.3)	9.2 (6.8-11.7)	33.6 (23.3-44.8)
Namibia	1.1 (0.0-3.9)	3.3 (0.4-10.1)	2.7 (0.2-9.7)
Niger	0.0	6.3 (4.7-8.8)	24.0 (17.3-32.6)
Nigeria	0.6 (0.3-1.1)	3.6 (2.4-4.6)	11.0 (9.3-12.9)
Congo	4.4 (2.0-8.1)	11.3 (7.2-16.9)	12.5 (6.9-19.9)
Rwanda	0.0	18.3 (10.6-28.4)	29.4 (20.8-38.1)
Senegal	0.0	3.7 (1.9-7.2)	5.4 (1.1-15.2)
Sierra Leone	0.7 (0.2-1.6)	14.5 (12.4-17.0)	35.0 (30.2-40.0)
Somalia	0.3 (0.0-1.4)	0.6 (0.0-3.7)	0.6 (0.0-4.1)
South Africa	2.7 (1.8-4.4)	4.3 (2.9-7.1)	7.5 (5.0-12.4)
South Sudan	3.5 (2.3-5.7)	14.0 (9.8-20.5)	27.7 (19.7-39.2)
Sudan	2.9 (1.9-4.7)	6.3 (4.4-9.6)	10.6 (7.4-16.3)
Swaziland	3.3 (2.3-5.2)	6.2 (4.3-9.4)	9.0 (6.1-14.3)
Tanzania	5.5 (3.5-7.5)	40.4 (34.6-46.3)	46.1 (37.5-54.8)
Togo	0.4 (0.1-1.1)	8.3 (6.5-10.1)	9.8 (7.1-13.1)
Uganda	0.6 (0.4-1.0)	25.2 (21.7-30.1)	70.2 (65.6-74.5)
Zambia	3.2 (1.5-6.2)	31.3 (26.8-35.6)	44.6 (39.4-50.5)
Zimbabwe	0.0	1.0 (0.1-7.7)	1.5 (0.0-20.1)

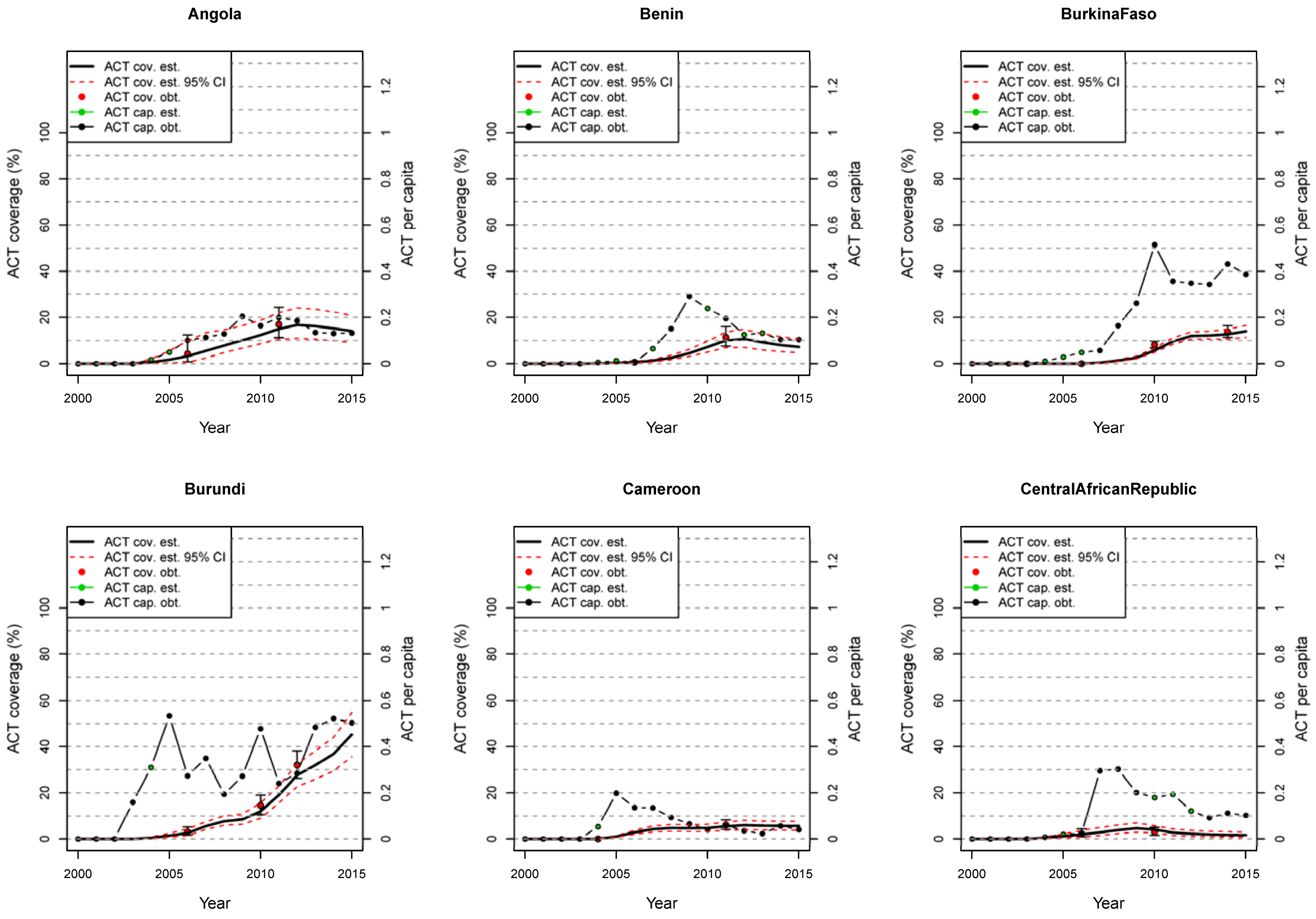
Angola
Benin
BurkinaFaso
Burundi
Cameroon
CentralAfricanRepublic
ACT cov. est.
ACT cov. est. 95% CI
ACT cov. obt.
ACT cap. est.
ACT cap. obt.
ACT coverage (%)
ACT per capita
Year
0
20
40
60
80
100
0.2
0.4
0.6
0.8
1
1.2
2000
2005
2010
2015

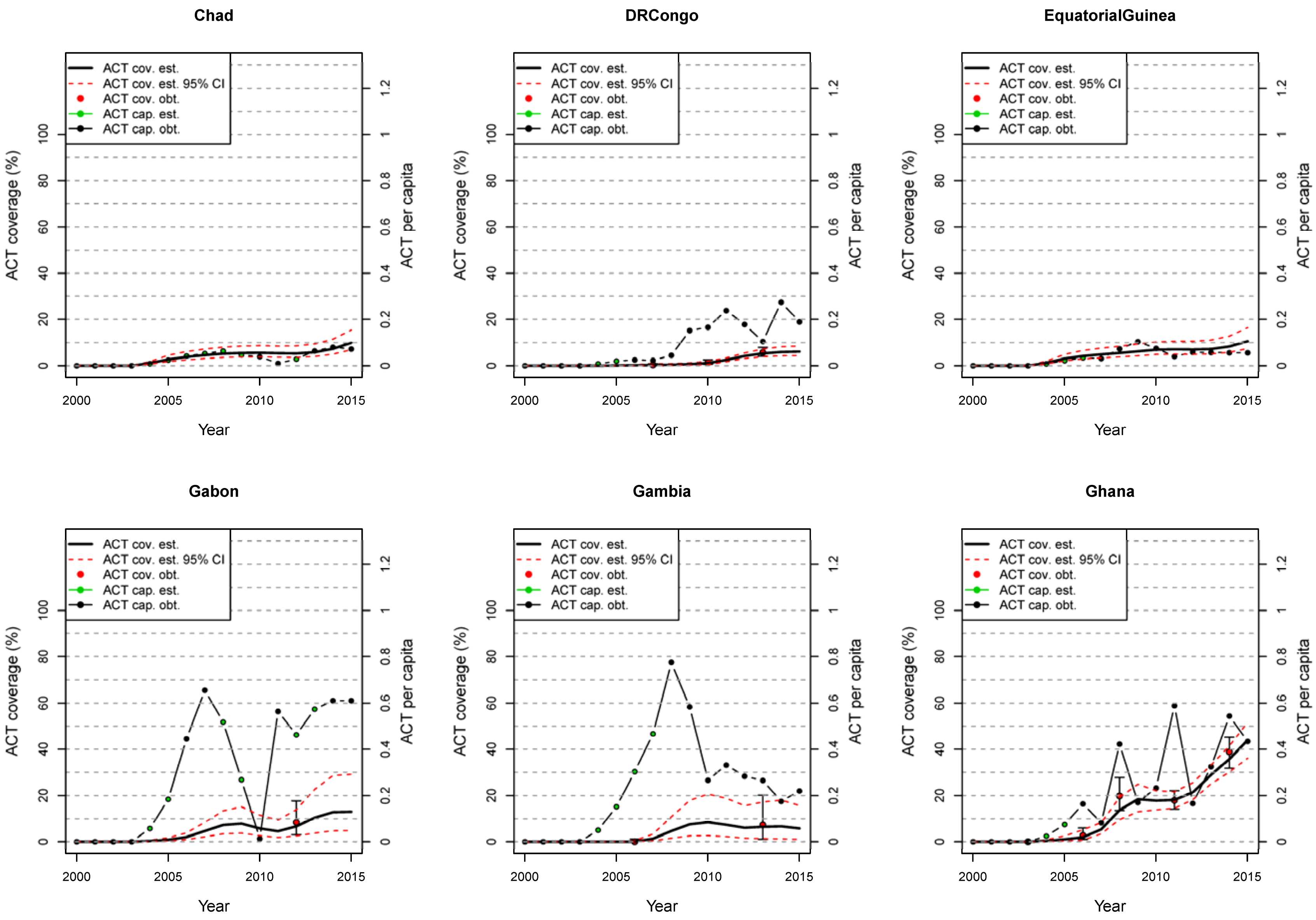
Chad
DRCongo
EquatorialGuinea
Gabon
Gambia
Ghana
ACT cov. est.
ACT cov. est. 95% CI
ACT cov. obt.
ACT cap. est.
ACT cap. obt.
ACT coverage (%)
ACT per capita
Year
0
20
40
60
80
100
0.2
0.4
0.6
0.8
1
1.2
2000
2005
2010
2015

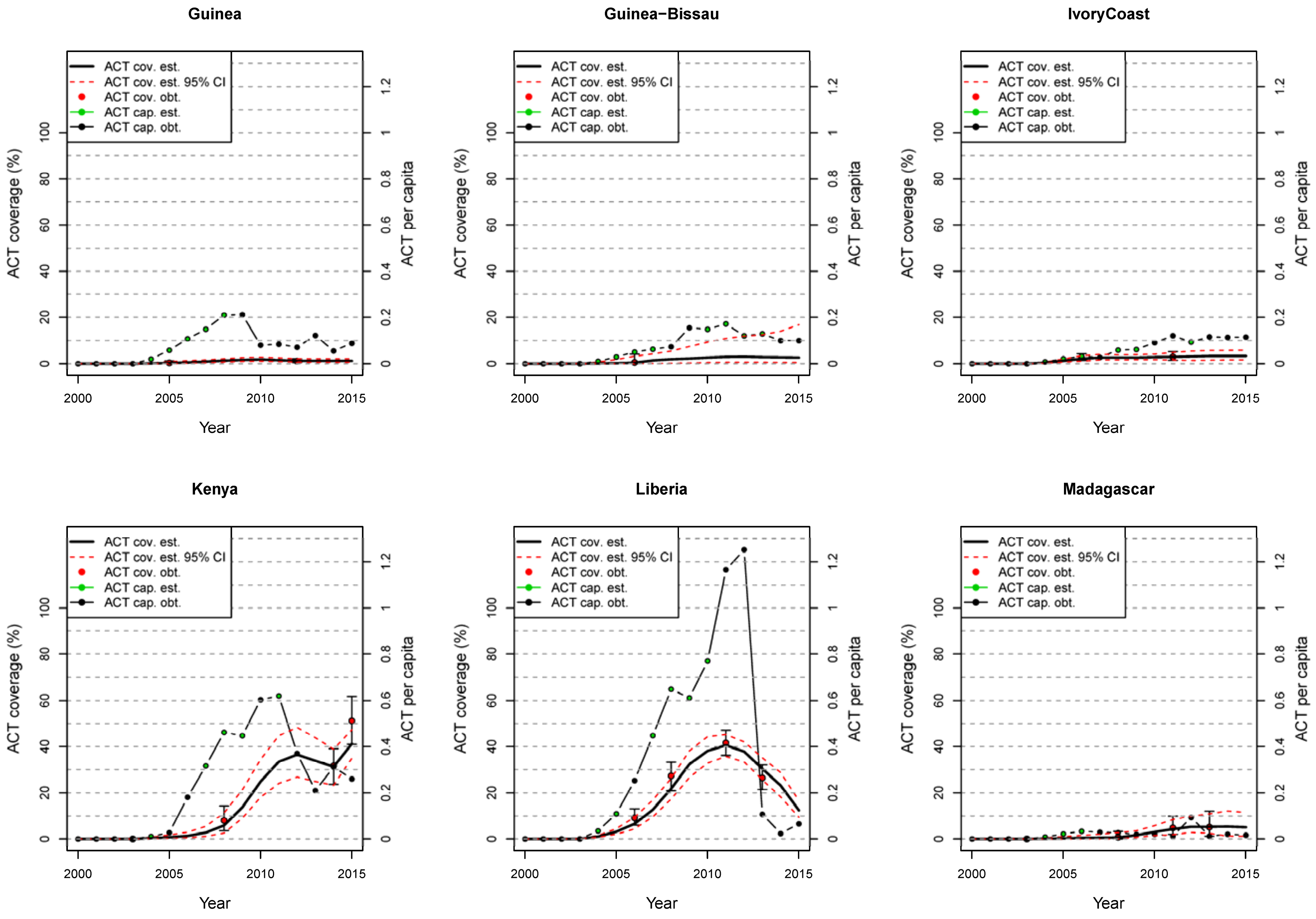
Guinea
Guinea−Bissau
IvoryCoast
Kenya
Liberia
Madagascar
ACT cov. est.
ACT cov. est. 95% CI
ACT cov. obt.
ACT cap. est.
ACT cap. obt.
ACT coverage (%)
ACT per capita
Year
0
20
40
60
80
100
0.2
0.4
0.6
0.8
1
1.2
2000
2005
2010
2015

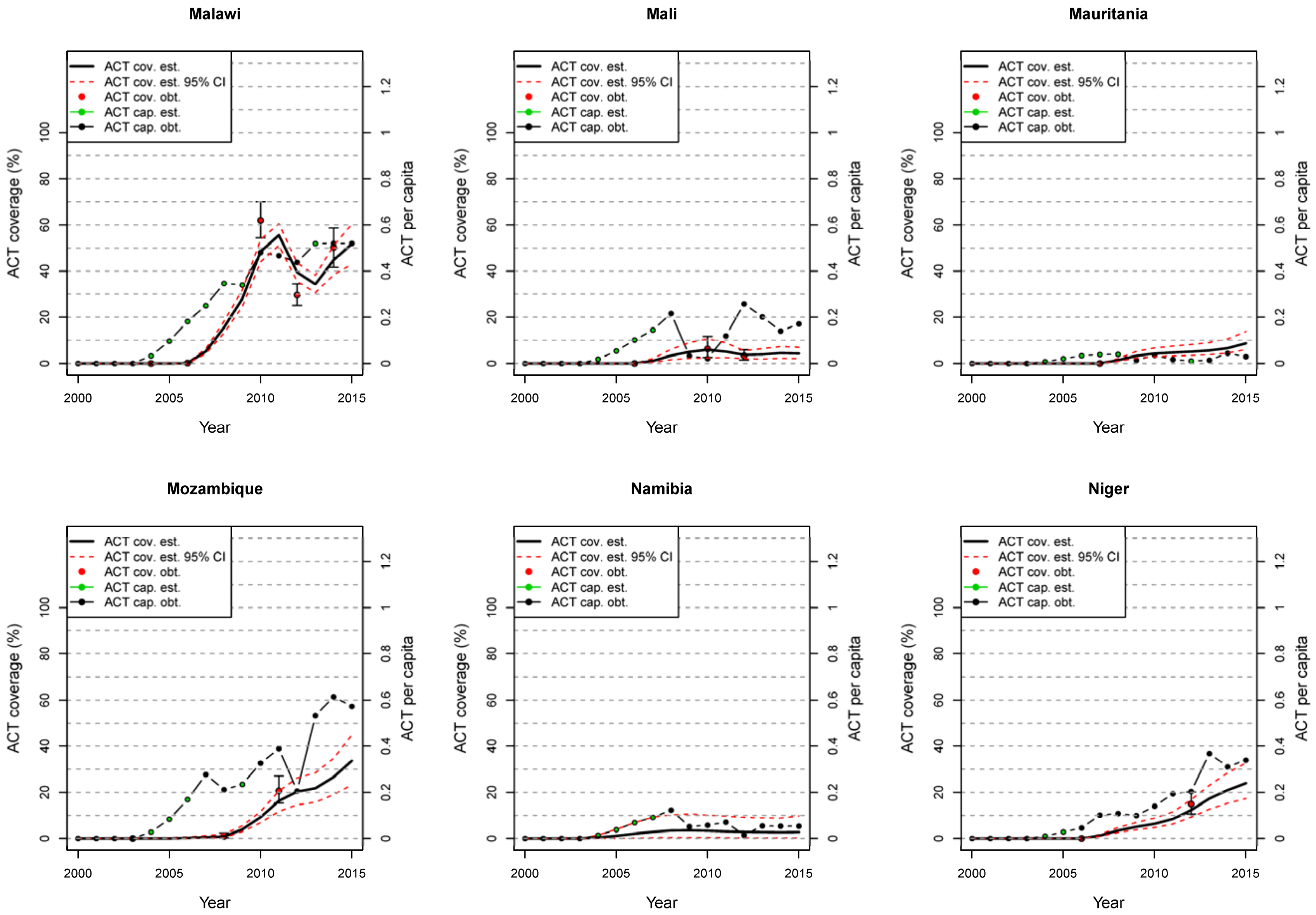
Malawi
Mali
Mauritania
Mozambique
Namibia
Niger
ACT cov. est.
ACT cov. est. 95% CI
ACT cov. obt.
ACT cap. est.
ACT cap. obt.
ACT coverage (%)
ACT per capita
Year
0
20
40
60
80
100
0.2
0.4
0.6
0.8
1
1.2
2000
2005
2010
2015

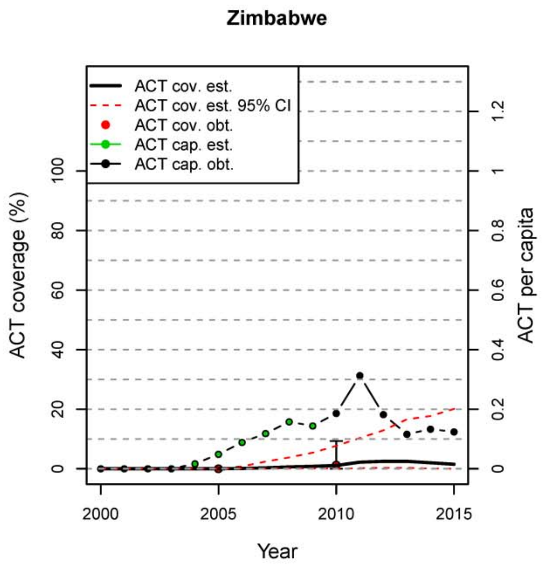

Zimbabwe
ACT cov. est.
ACT cov. est. 95% CI
ACT cov. obt.
ACT cap. est.
ACT cap. obt.
ACT coverage (%)
ACT per capita
Year
0
20
40
60
80
100
0
0.2
0.4
0.6
0.8
1
1.2
2000
2005
2010
2015

Meta-regression of country datasets

Multivariable regression models predicting ACT treatment in children <5 with fever and *P. falciparum* infection (RDT+) were conducted on each country level dataset including the predictors age (greater than/less than 2 years of age), caregiver education (any vs. none), wealth (above vs. below country median), household ITN ownership, $\textit{Pf}\text{PR}_{2\text{-}10}$ mean, and urban vs. rural. These models were run on a random set of 100 out of the 1000 RDT+ child level predictions. For each country dataset and parameter from this model, the median coefficient and standard error were extracted and entered into separate random effects meta-regressions, using the Dersimion and Laird method [8]. A separate set of regressions was run on only those children who sought care, with the additional parameter type of treatment location (public vs. private). Meta-analyses and plots were conducted using the *metafor* package in R.

Figures 11-24 below depict forest plots for meta-analyses on individual predictors of ACT treatment in children <5 with *P. falciparum* infection. For all plots, labels represent concatenated country code, year, and survey type (DHS=0, MIS=1, MICS=2).

Figure 11. Forest plot of country data-set specific odds ratios on the outcome of ACT treatment, for age (greater than two years of age vs. less than or equal to two years of age).

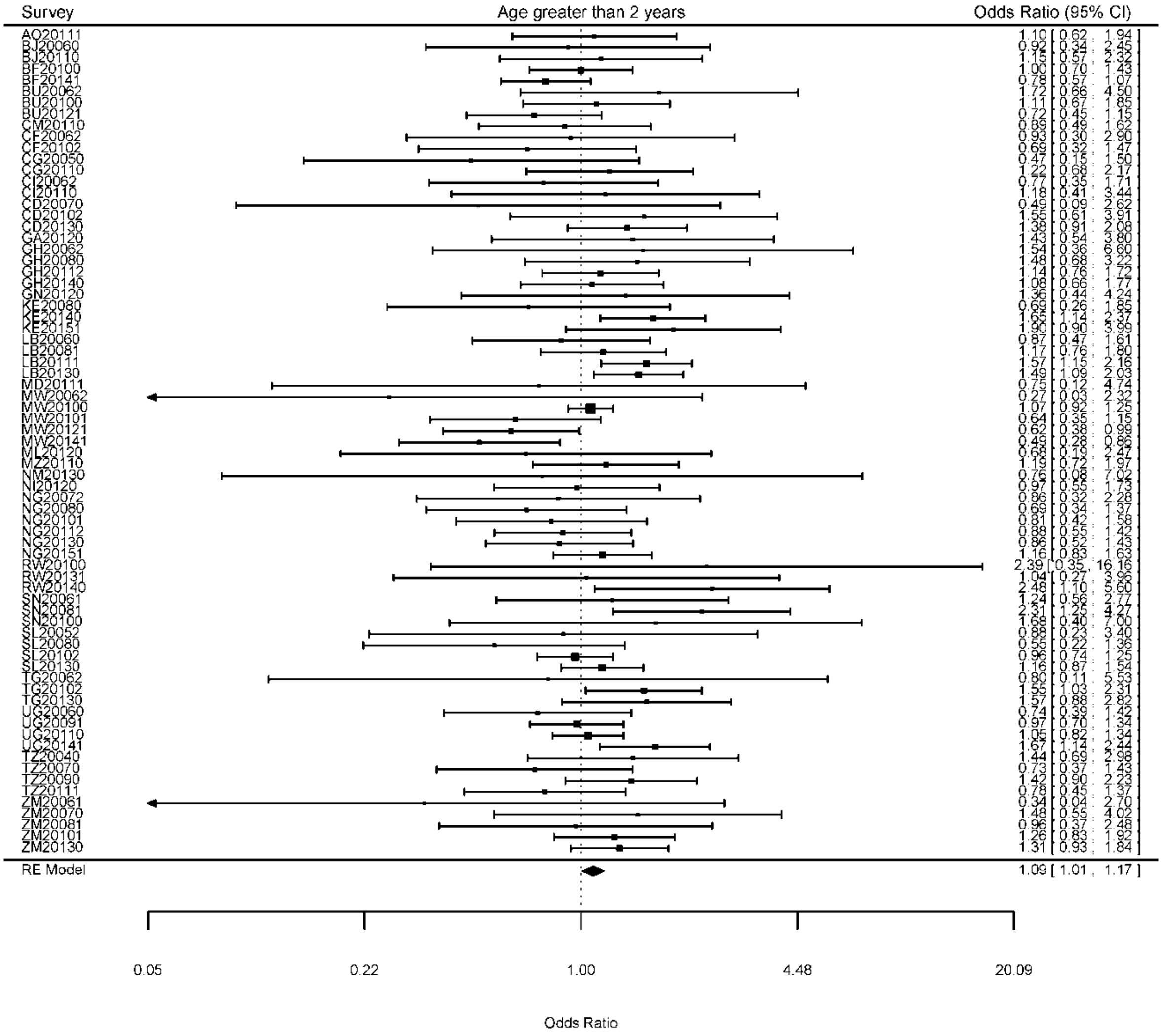

Figure 12. Forest plot of country data-set specific odds ratios for caregiver's education (any vs. none).

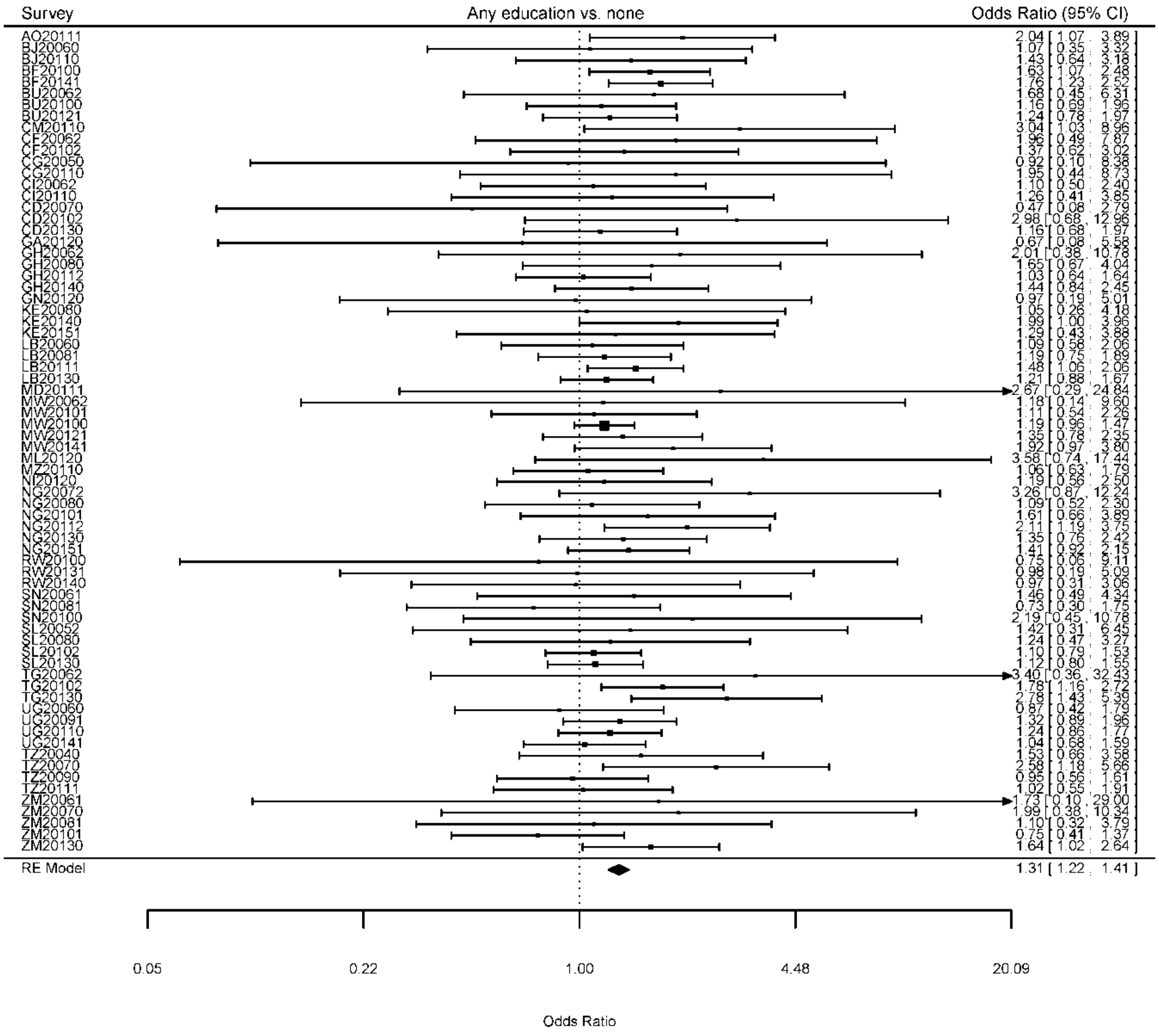

Figure 13. Forest plot of country data-set specific odds ratios for household wealth (above vs. below country median).

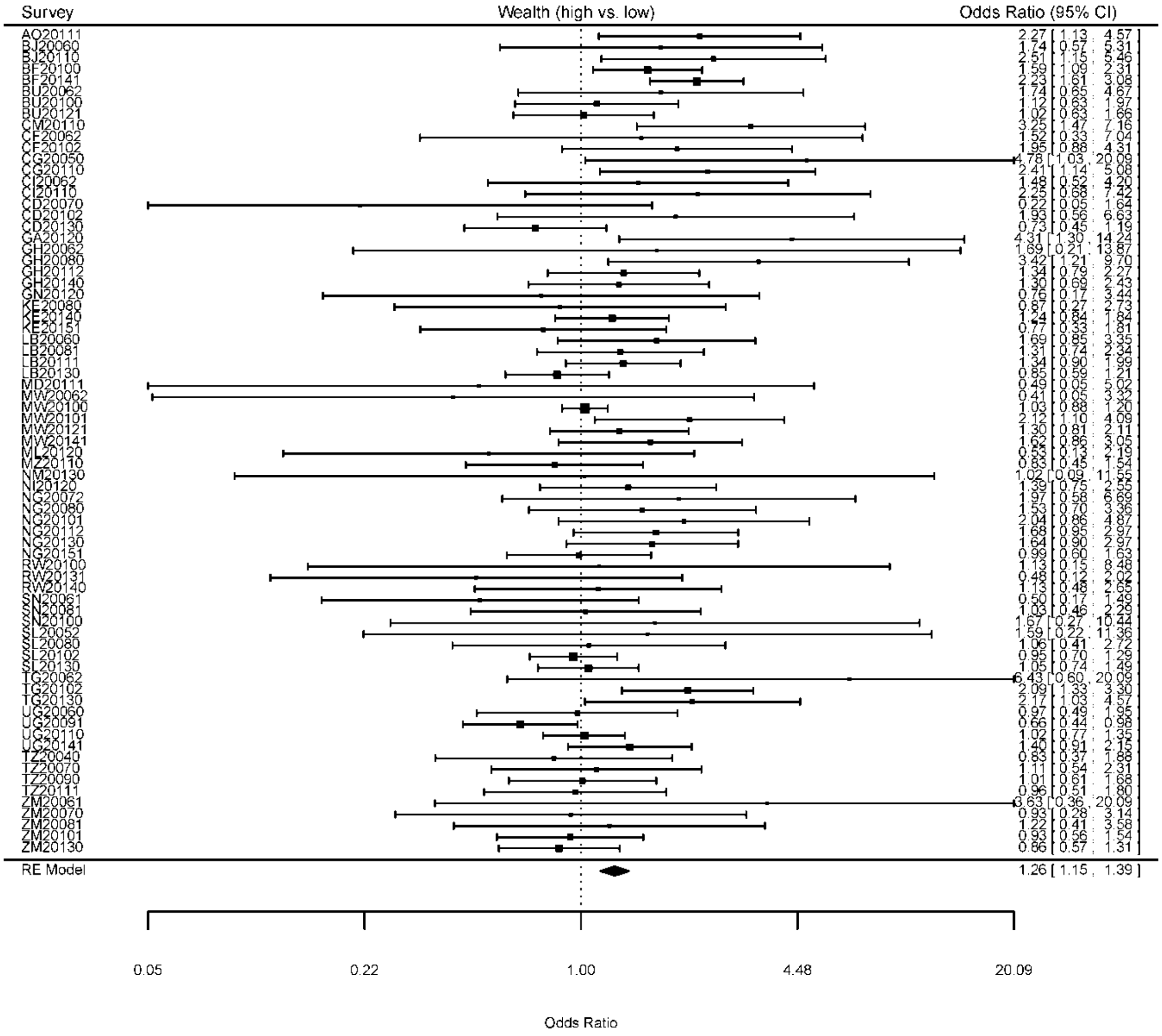

Figure 14. Forest plot of country data-set specific odds ratios for household ITN ownership (any ITN vs. none).

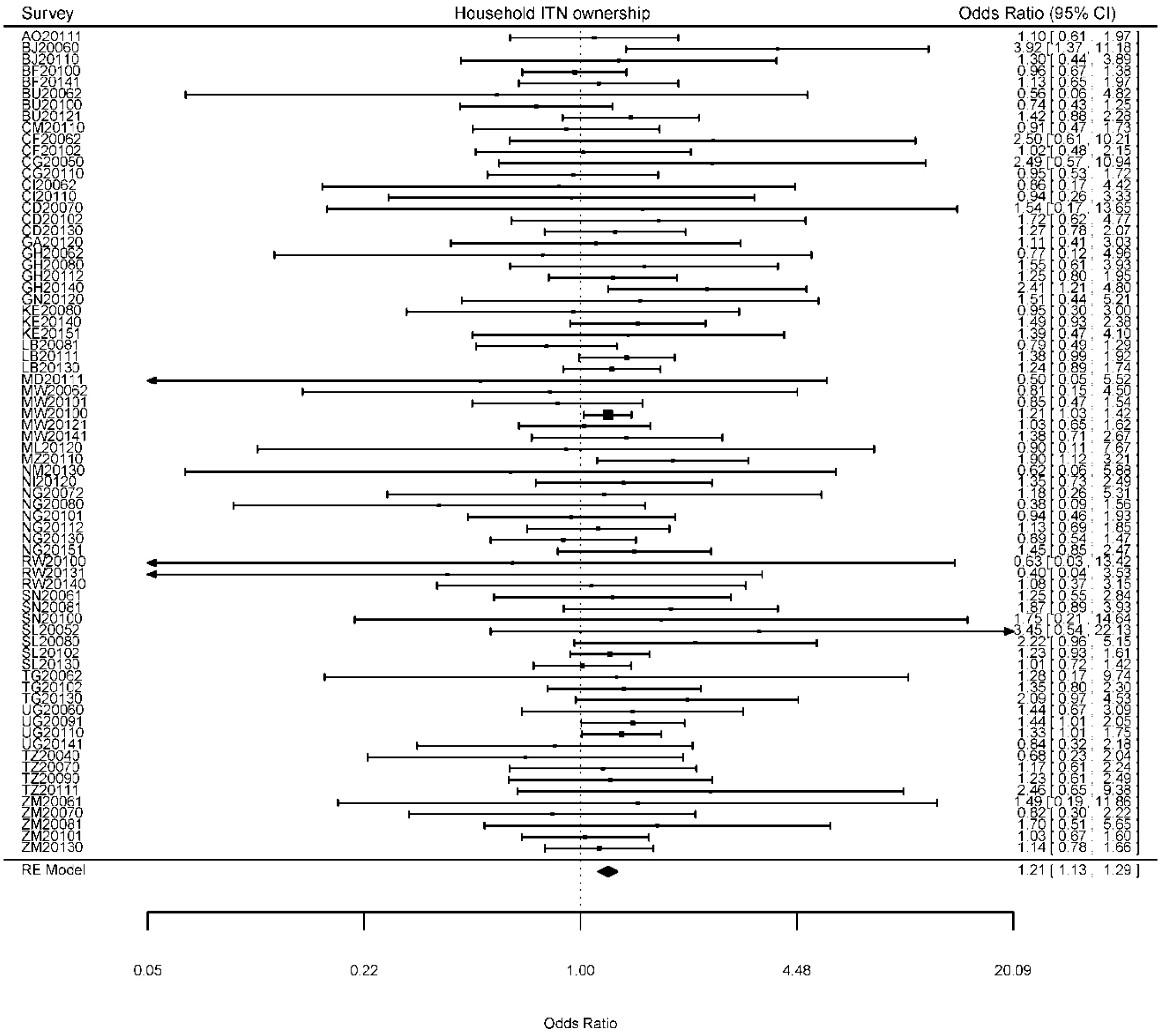

Figure 15. Forest plot of country data-set specific odds ratios for urban residence (urban vs. rural).

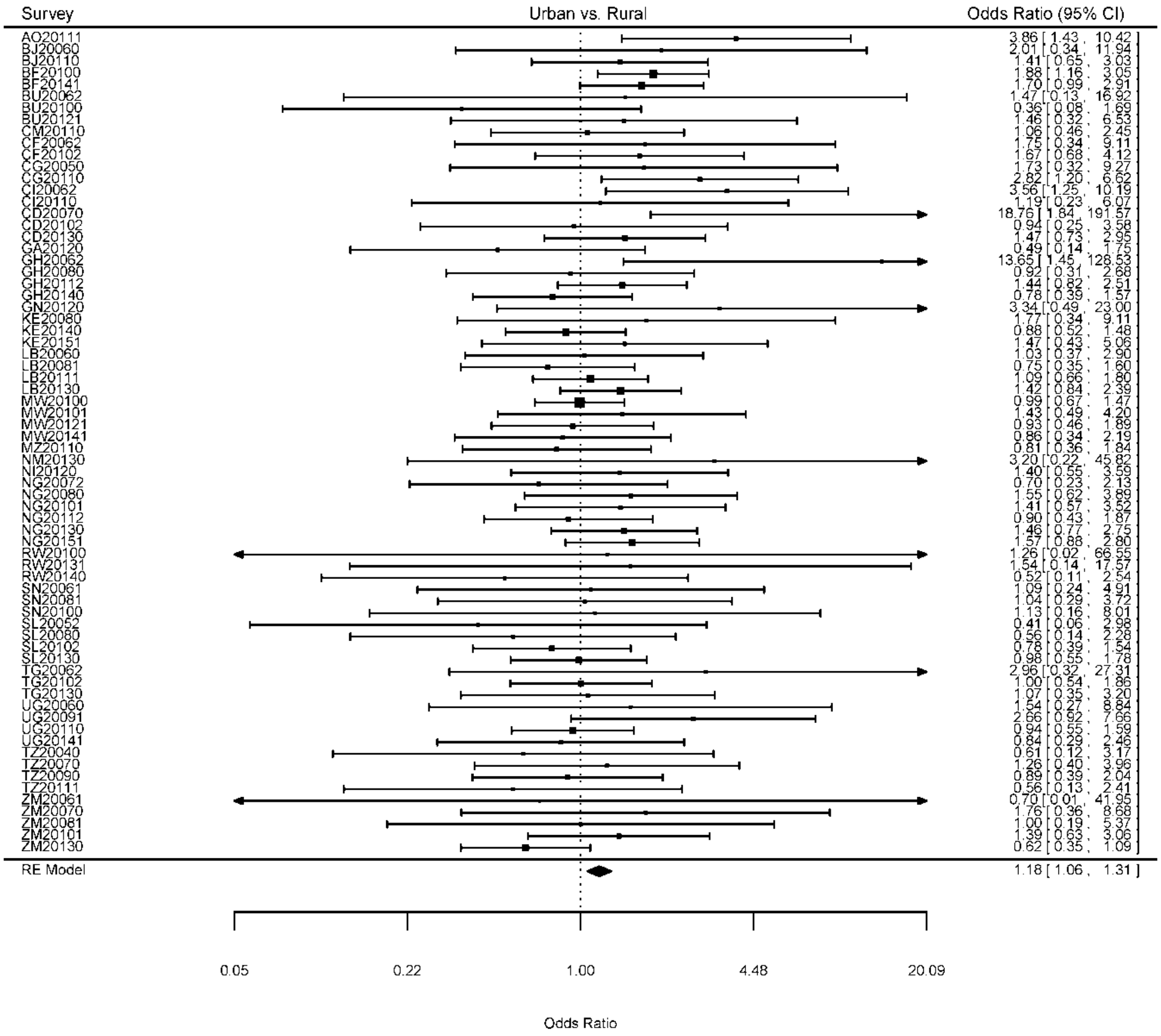

Figure 16. Forest plot of country data-set specific odds ratios for mean (logit-transformed) *Pf*PR$_{2\text{-}10}$.

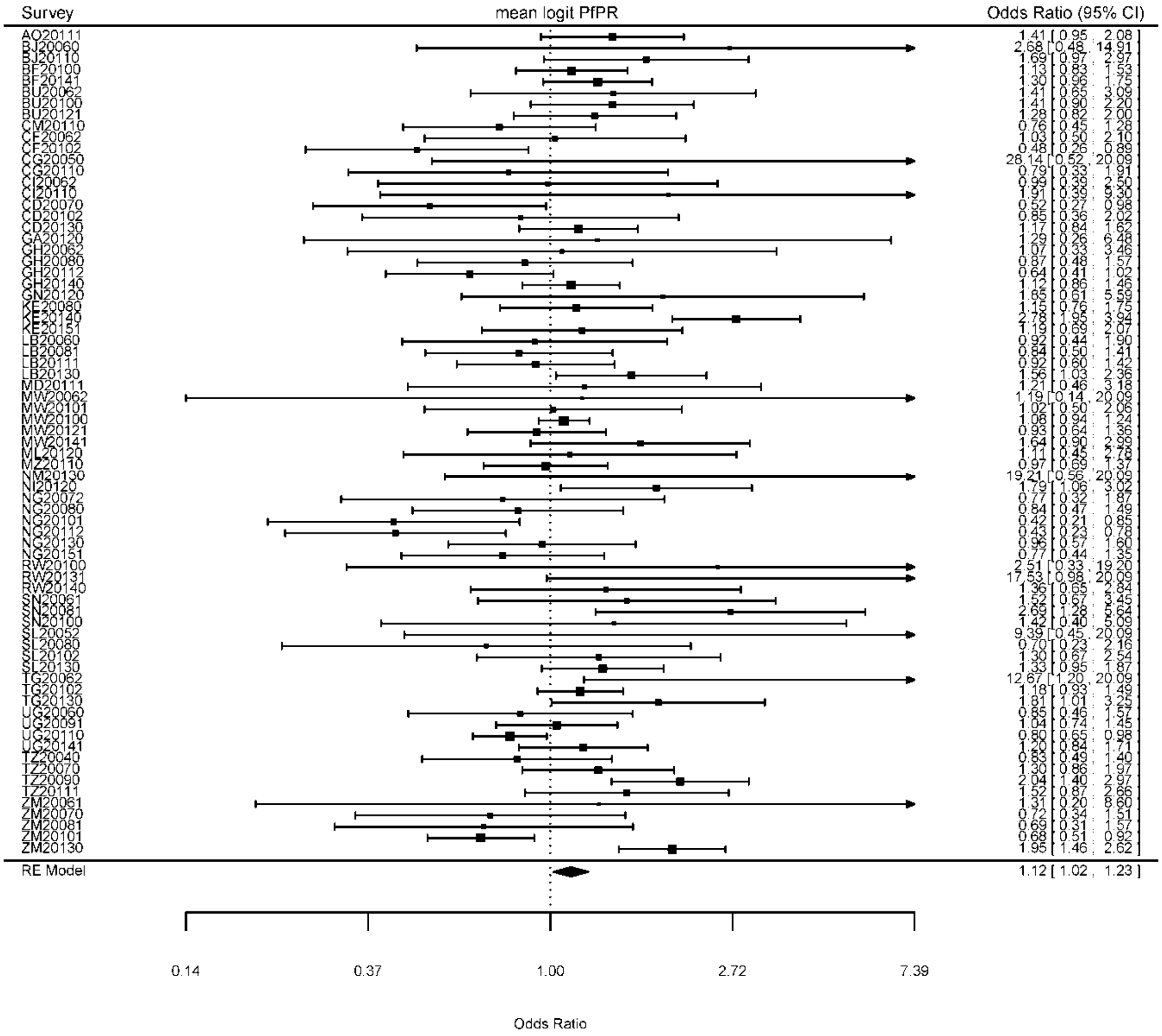

Figure 17. Forest plot of country data-set specific odds ratios for age (greater than 2 years old vs. less than or equal to 2 years old), among those children who sought care.

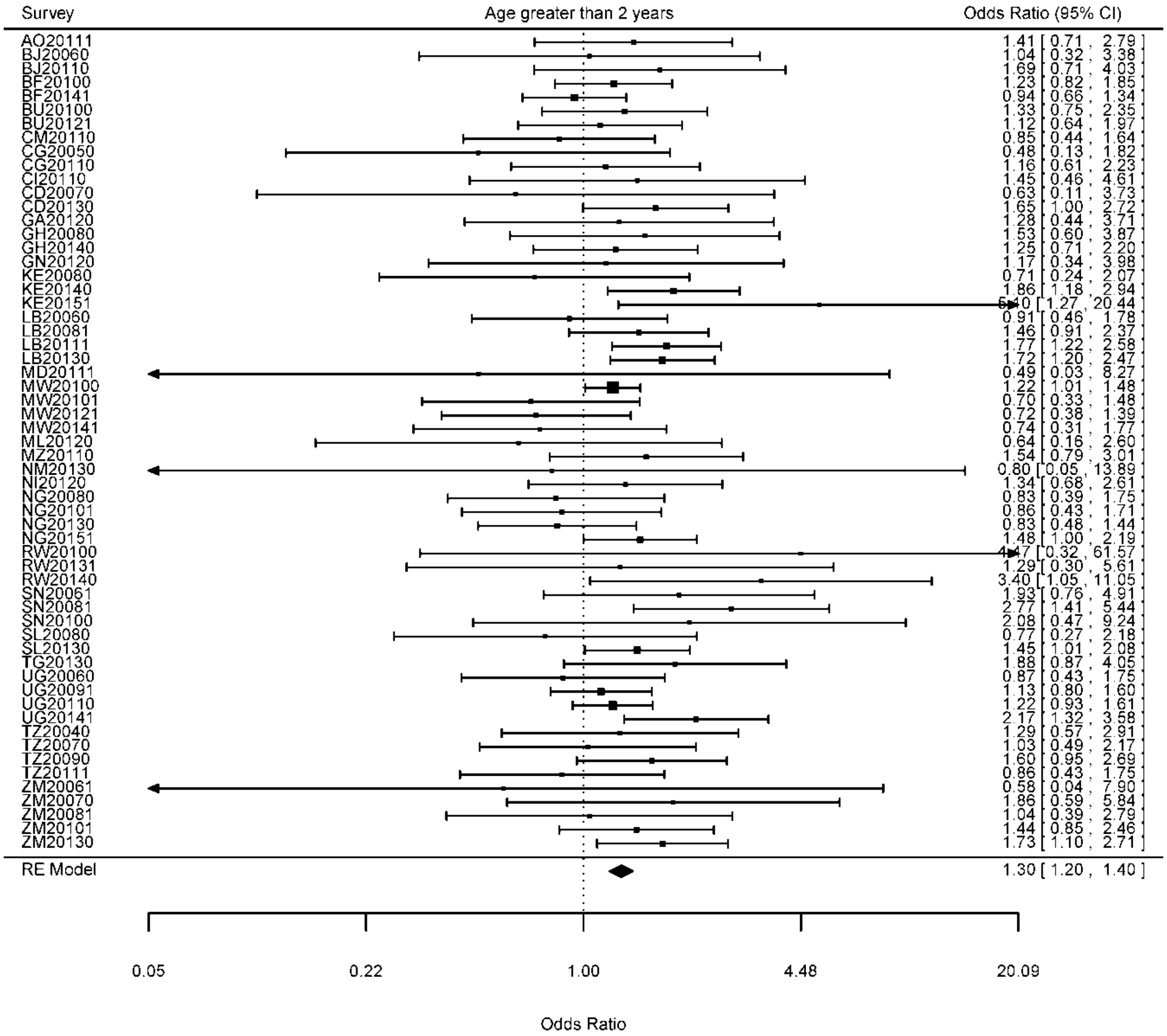

Figure 18. Forest plot of country data-set specific odds ratios for caregiver's education (any vs. none), among those who sought care.

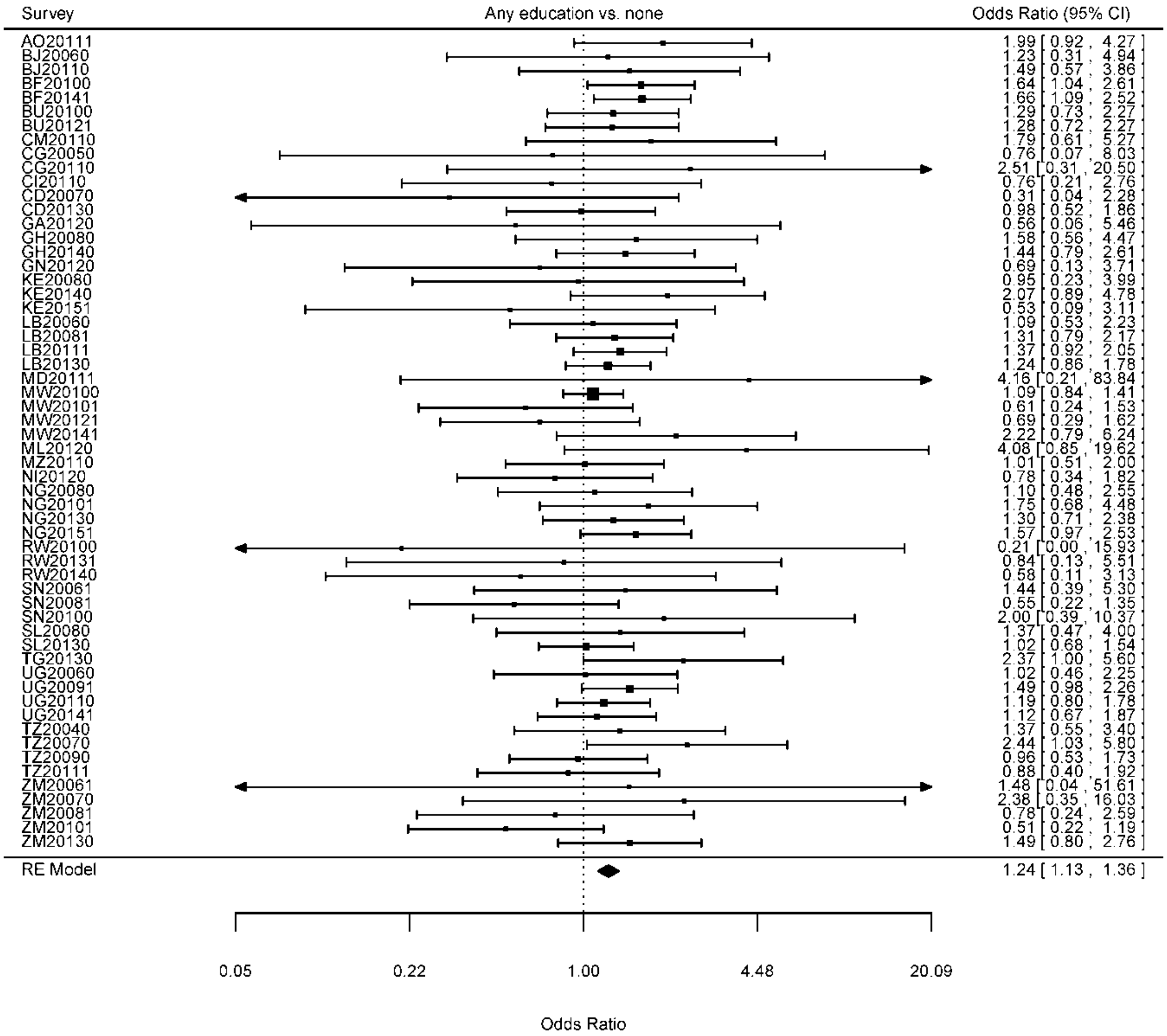

Figure 19. Forest plot of country data-set specific odds ratios for household wealth (above vs. below country median), among those who sought care.

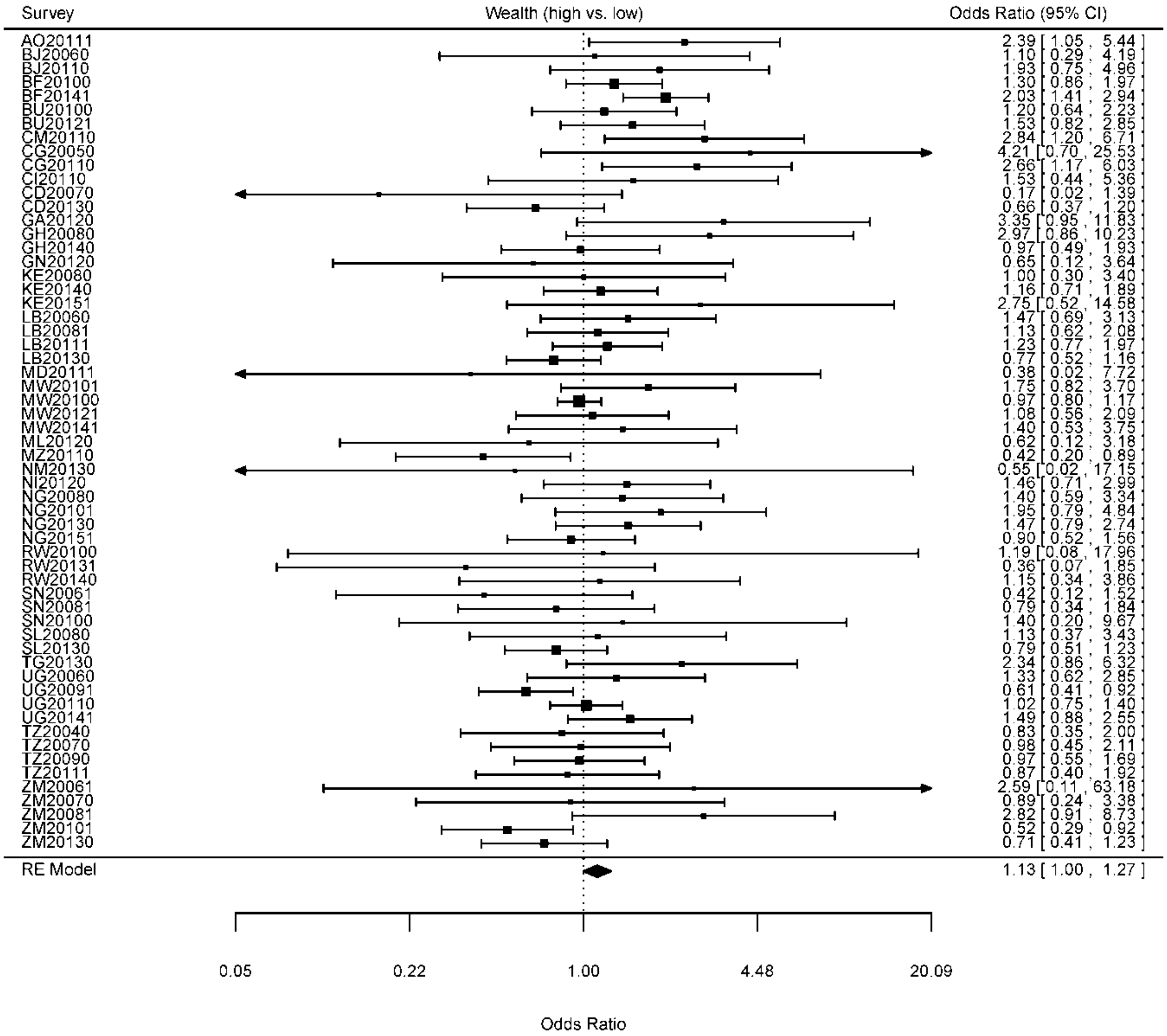

Figure 20. Forest plot of country data-set specific odds ratios for household ITN ownership (any ITN vs. none), among those who sought care.

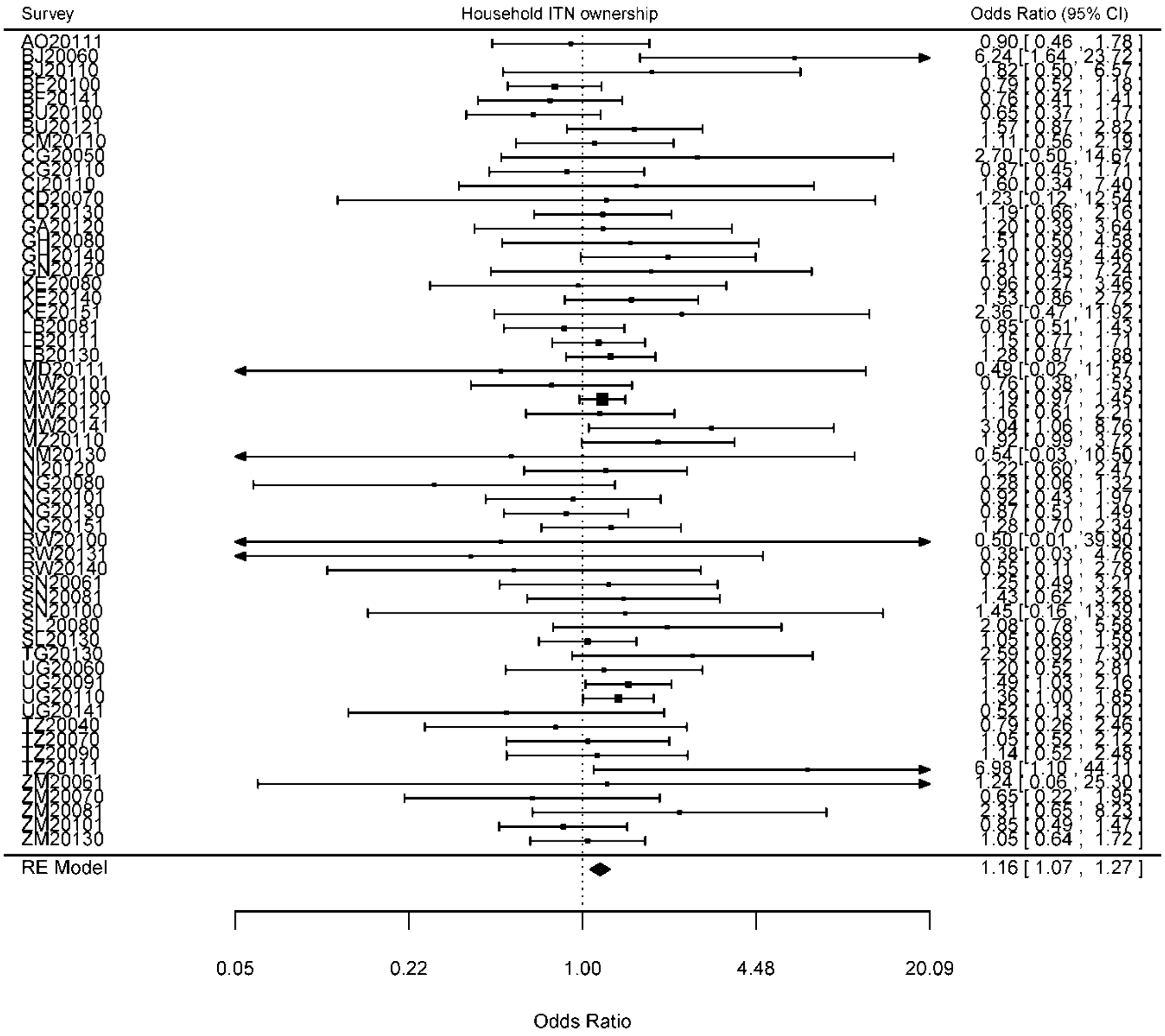

Figure 21. Forest plot of country data-set specific odds ratios for urban residence (urban vs. rural), among those who sought care.

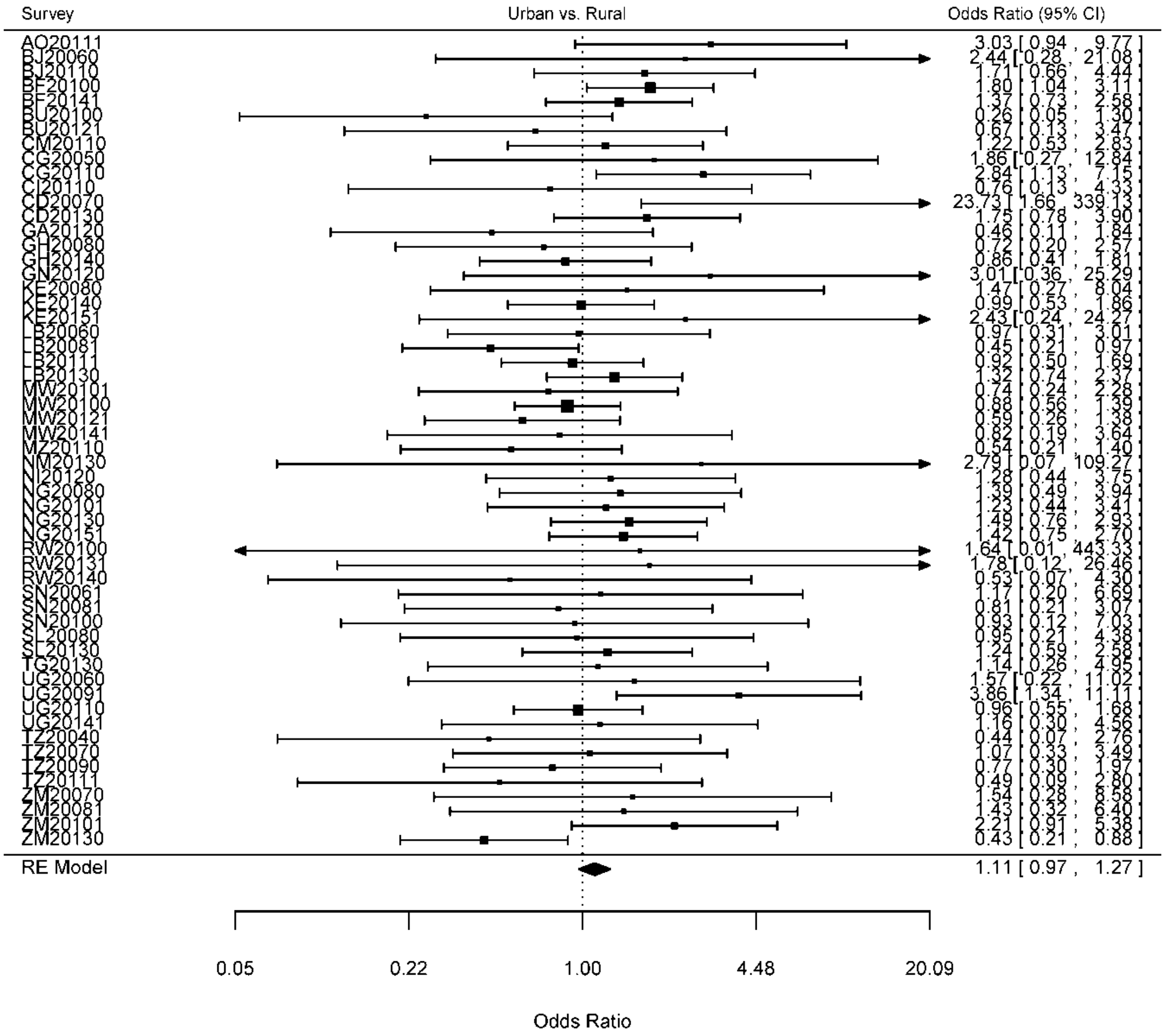

Figure 22. Forest plot of country data-set specific odds ratios for mean (logit-transformed) *Pf*PR$_{2\text{-}10}$, among those who sought care.

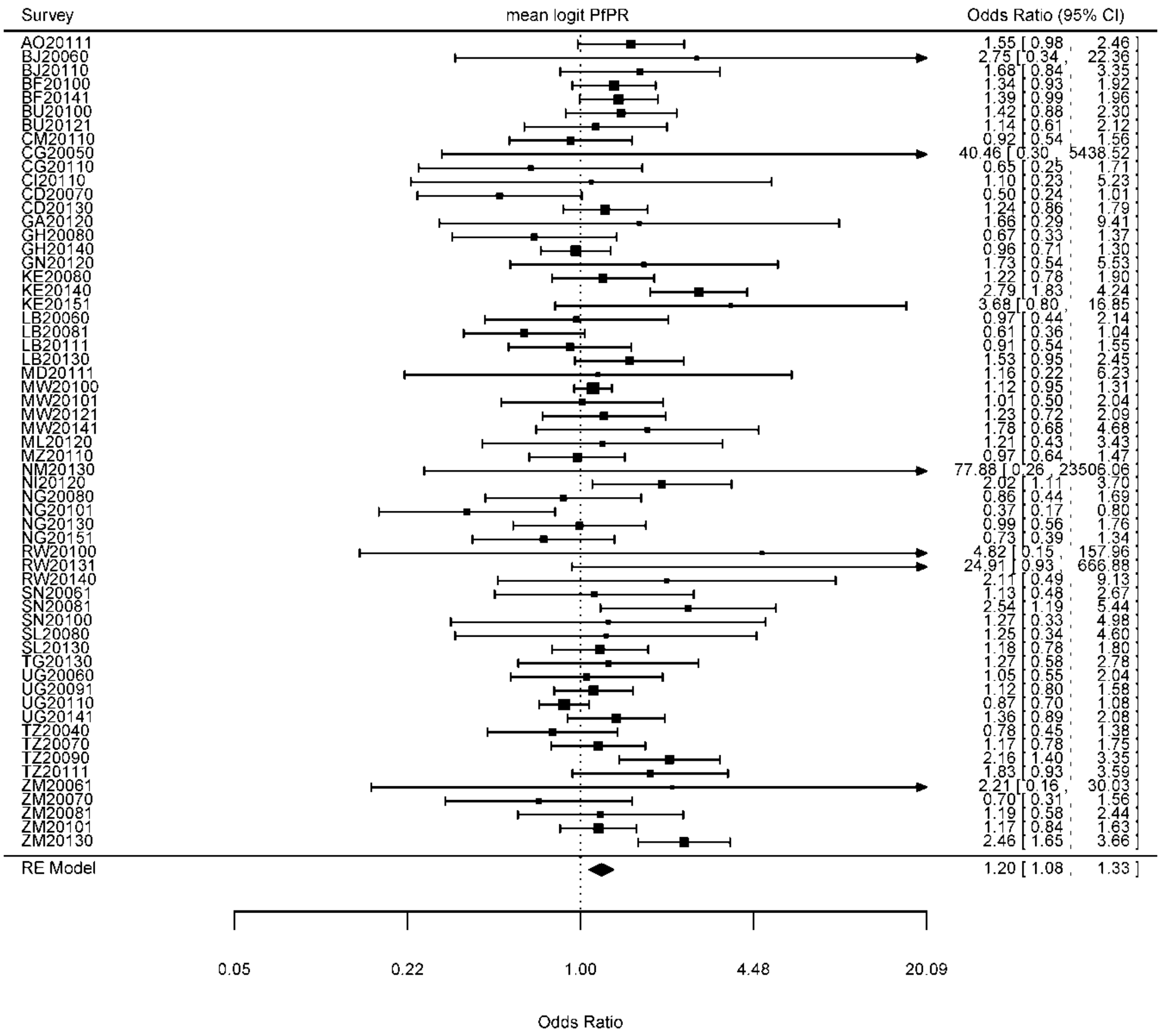

Figure 23. Forest plot of country data-set specific odds ratios for treatment seeking at public vs. private providers.

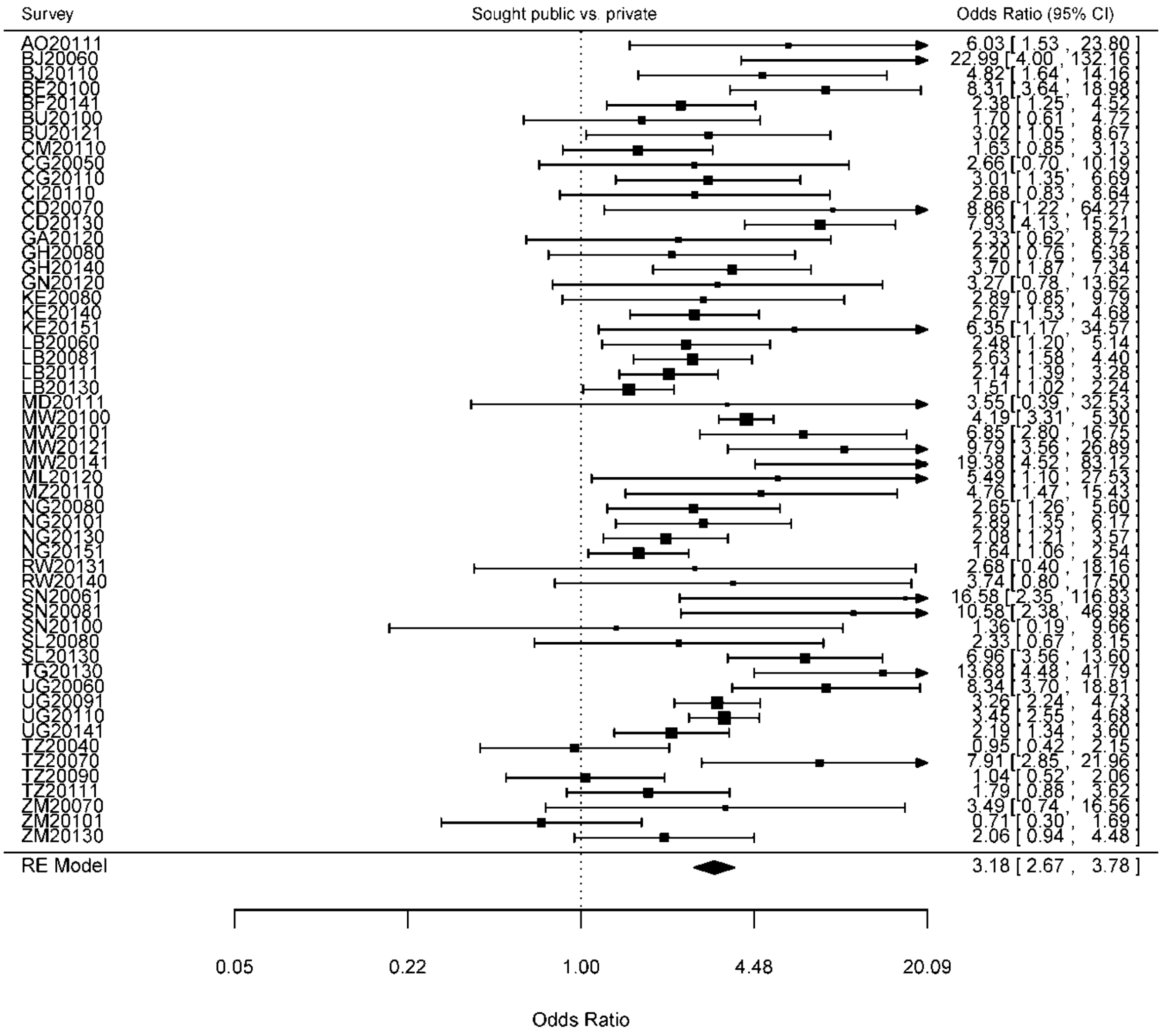

Figure 24. Forest plot of country data-set specific odds ratios for RDT+ vs. RDT-.

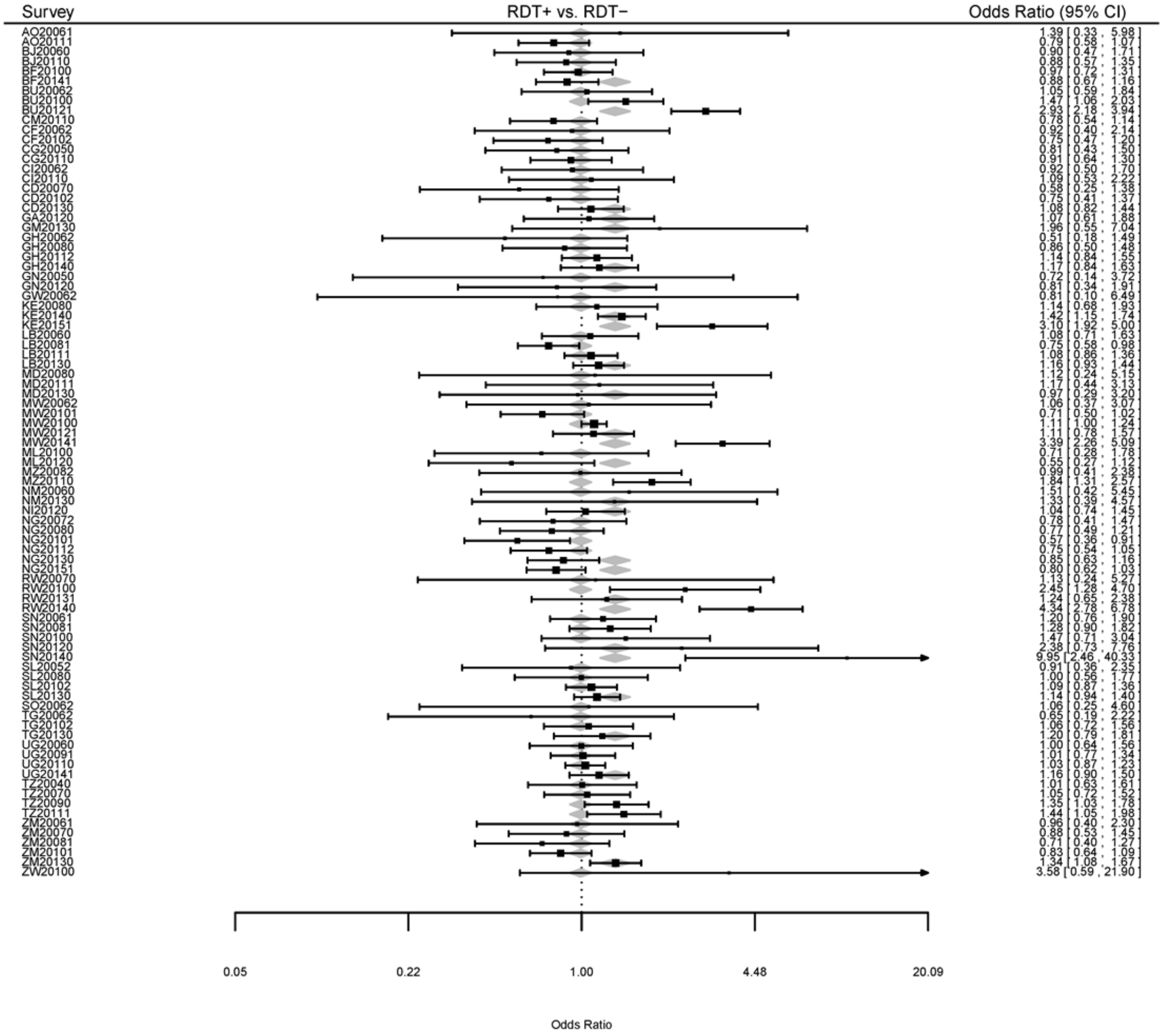

Ta ble 4. STROBE checklist.

**STROBE Checklist for cross-sectional studies**	**Item No.**	**Recommendations**	**Location addressed in manuscript**
**Title and abstract**	1	(*a*) Indicate the study's design with a commonly used term in the title or the abstract<br>(*b*) Provide in the abstract an informative and balanced summary of what was done and what was found	Abstract
**Introduction**			
Background	2	Explain the scientific background and rationale for the investigation being reported	Introduction
Objectives	3	State specific objectives, including any pre-specified hypotheses	Introduction
**Methods**			
Study design	4	Present key elements of study design early in the paper	Methods: "Statistical analysis" subheading
Setting	5	Describe the setting, locations, and relevant dates, including periods of recruitment,<br>exposure, follow-up, and data collection	Methods: "Data sources" subheading
Participants	6	Give the eligibility criteria, and the sources and methods of selection of<br>participants	Methods: "Data sources" and "Definitions" subheading
Variables	7	Clearly define all outcomes, exposures, predictors, potential confounders, and effect modifiers. Give diagnostic criteria, if applicable	Methods: "Definitions" subheading
Data sources/measurement	8	For each variable of interest, give sources of data and details of methods of assessment (measurement). Describe comparability of assessment methods if there is more than one group	Methods: "Data sources" subheading
Bias	9	Describe any efforts to address potential sources of bias	Methods: "Model validation" subheading
Study size	10	Explain how the study size was arrived at	Methods: "Data sources" subheading
Quantitative variables	11	Explain how quantitative variables were handled in the analyses. If applicable, describe which groupings were chosen and why	Methods: "Definitions" subheading
Statistical methods	12	(*a*) Describe all statistical methods, including those used to control for confounding<br>(*b*) Describe any methods used to examine subgroups and interactions<br>(*c*) Explain how missing data were addressed<br>(*d*) If applicable, describe analytical methods taking account of sampling strategy<br>(*e*) Describe any sensitivity analyses	Methods: "Statistical analysis", "National-level coverage estimates", "Meta-regression of factors…" subheadings
**Results**			
Participants	13	(a) Report numbers of individuals at each stage of study—eg numbers potentially eligible, examined for eligibility, confirmed eligible, included in the study, completing follow-up, and analysed<br>(b) Give reasons for non-participation at each stage<br>(c) Consider use of a flow diagram	Results and Supplementary Material
Descriptive data	14	(a) Give characteristics of study participants (eg demographic, clinical, social) and information on	Results and Supplementary Material

		exposures and potential confounders<br>(b) Indicate number of participants with missing data for each variable of interest	
Outcome data	15	Report numbers of outcome events or summary measures	Results and Supplementary Material
Main results	16	(*a*) Give unadjusted estimates and, if applicable, confounder-adjusted estimates and their precision (eg, 95% confidence interval). Make clear which confounders were adjusted for and why they were included<br>(*b*) Report category boundaries when continuous variables were categorized<br>(*c*) If relevant, consider translating estimates of relative risk into absolute risk for a meaningful time period	Results and Supplementary Material
Other analyses	17	Report other analyses done—eg analyses of subgroups and interactions, and sensitivity analyses	Results and Supplementary Material
**Discussion**			
Key results	18	Summarise key results with reference to study objectives	Discussion
Limitations	19	Discuss limitations of the study, taking into account sources of potential bias or imprecision. Discuss both direction and magnitude of any potential bias	Discussion
Interpretation	20	Give a cautious overall interpretation of results considering objectives, limitations, multiplicity of analyses, results from similar studies, and other relevant evidence	Discussion
Generalisability	21	Discuss the generalisability (external validity) of the study results	Discussion
**Other information**			
Funding	22	Give the source of funding and the role of the funders for the present study and, if applicable, for the original study on which the present article is based	"Role of the funding source" subheading and Acknowledgements

References

1. MAP, *Malaria Atlas Project*. 2015, University of Oxford: Oxford, England.
2. Keating, J., et al., *Plasmodium falciparum parasite infection prevalence from a household survey in Zambia using microscopy and a rapid diagnostic test: implications for monitoring and evaluation.* Acta Trop, 2009. **112**: p. 277 - 282.
3. Willcox, M.L., et al., *Rapid diagnostic tests for the home-based management of malaria, in a high-transmission area.* Ann Trop Med Parasitol, 2009. **103**(1): p. 3-16.
4. Aydin-Schmidt, B., et al., *Usefulness of Plasmodium falciparum-specific rapid diagnostic tests for assessment of parasite clearance and detection of recurrent infections after artemisinin-based combination therapy*. Malar J, 2013. **12**: p. 349.
5. WHO, *World Malaria Report 2014*, WHO, Editor. 2014: Geneva.
6. Worldpop, *http://www.worldpop.org.uk/*. 2015.
7. WHO, *World Malaria Report 2013*. 2013.
8. Dersimonian R, L.N., *Meta-analysis in clinical trials.* Control Clin Trials, 1986. **7**(3): p. 177-88.
